# Class XI Myosins Contribute to Auxin Response and Senescence-Induced Cell Death in *Arabidopsis*

**DOI:** 10.3389/fpls.2018.01570

**Published:** 2018-11-27

**Authors:** Eve-Ly Ojangu, Birger Ilau, Krista Tanner, Kristiina Talts, Eliis Ihoma, Valerian V. Dolja, Heiti Paves, Erkki Truve

**Affiliations:** ^1^Department of Chemistry and Biotechnology, Tallinn University of Technology, Tallinn, Estonia; ^2^Department of Botany and Plant Pathology, Oregon State University, Corvallis, OR, United States

**Keywords:** *Arabidopsis*, myosin XI, auxin response, PIN1, flower development, senescence, SAG13, anthocyanins

## Abstract

The integrity and dynamics of actin cytoskeleton is necessary not only for plant cell architecture but also for membrane trafficking-mediated processes such as polar auxin transport, senescence, and cell death. In *Arabidopsis*, the inactivation of actin-based molecular motors, class XI myosins, affects the membrane trafficking and integrity of actin cytoskeleton, and thus causes defective plant growth and morphology, altered lifespan and reduced fertility. To evaluate the potential contribution of class XI myosins to the auxin response, senescence and cell death, we followed the flower and leaf development in the triple gene knockout mutant *xi1 xi2 xik* (3KO) and in rescued line stably expressing myosin XI-K:YFP (3KOR). Assessing the development of primary inflorescence shoots we found that the 3KO plants produced more axillary branches. Exploiting the auxin-dependent reporters DR5::GUS and IAA2::GUS, a significant reduction in auxin responsiveness was found throughout the development of the 3KO plants. Examination of the flower development of the plants stably expressing the auxin transporter PIN1::PIN1-GFP revealed partial loss of PIN1 polarization in developing 3KO pistils. Surprisingly, the stable expression of PIN1::PIN1-GFP significantly enhanced the semi-sterile phenotype of the 3KO plants. Further we investigated the localization of myosin XI-K:YFP in the 3KOR floral organs and revealed its expression pattern in floral primordia, developing pistils, and anther filaments. Interestingly, the XI-K:YFP and PIN1::PIN1-GFP shared partially overlapping but distinct expression patterns throughout floral development. Assessing the foliar development of the 3KO plants revealed increased rosette leaf production with signs of premature yellowing. Symptoms of the premature senescence correlated with massive loss of chlorophyll, increased cell death, early plasmolysis of epidermal cells, and strong up-regulation of the stress-inducible senescence-associated gene *SAG13* in 3KO plants. Simultaneously, the reduced auxin responsiveness and premature leaf senescence were accompanied by significant anthocyanin accumulation in 3KO tissues. Collectively, our results provide genetic evidences that *Arabidopsis* class XI myosins arrange the flower morphogenesis and leaf longevity via contributing to auxin responses, leaf senescence, and cell death.

## Introduction

The dynamics of actin cytoskeleton, including filament assembly, disassembly and reorganization are regulated by diverse actin binding proteins (ABPs). Various genetic studies have shown that down-regulation of ABPs affects the organization and dynamics of the actin arrays in plant cells (Kandasamy et al., 2005; Henty-Ridilla et al., 2014; Li et al., 2014; Wu et al., 2015; Tang et al., 2017). Actin-dependent molecular motors, myosins, make up a one group of the ABPs that transport endomembranes and other macromolecular cargoes along actin filaments (AFs) in plant cells. In *Arabidopsis*, the cell elongation is affected significantly when multiple class XI myosins are simultaneously eliminated (Prokhnevsky et al., 2008; Peremyslov et al., 2010; Ueda et al., 2010; Ojangu et al., 2012; Cai et al., 2014; Madison et al., 2015; Scheuring et al., 2016; Talts et al., 2016; Abu-Abied et al., 2018). In particular, the stature of myosin XI single, double, triple, quadruple and quintuple knockout plants is decreased progressively along with the reduction in cell size (Ojangu et al., 2007, 2012; Peremyslov et al., 2008, 2010; Prokhnevsky et al., 2008; Ueda et al., 2010; Park and Nebenführ, 2013; Cai et al., 2014; Madison et al., 2015; Abu-Abied et al., 2018). At a subcellular level, myosin XI inactivation in triple knockout mutant *xi1 xi2 xik* (3KO) results in reorientation of prominent actin bundles, reduced dynamic behavior of actin arrays, changes in membrane trafficking and deceleration of cytoplasmic streaming (Peremyslov et al., 2010; Ueda et al., 2010; Park and Nebenführ, 2013; Cai et al., 2014; Scheuring et al., 2016). Thus, in the 3KO, cytoplasmic streaming is virtually arrested, the elongation of epidermal and other cell types is decreased and therefore plant size and fertility is affected (Peremyslov et al., 2010; Ueda et al., 2010; Ojangu et al., 2012; Cai et al., 2014; Scheuring et al., 2016).

In addition to being essential for cell integrity, actin cytoskeleton contributes to processes such as polar auxin transport (PAT) (Nick et al., 2009; Zhu and Geisler, 2015; Wu et al., 2015; Zhu et al., 2016; Eggenberger et al., 2017; Huang et al., 2017) and regulation of programmed cell death (PCD) (Kandasamy et al., 2005; Thomas et al., 2006; Breeze et al., 2011; Keech, 2011; Smertenko and Franklin-Tong, 2011).

Various genetic and pharmacological studies have revealed tight interplay between auxin signaling and actin cytoskeleton. On the one hand, the patterning of actin arrays is modulated by auxin; on the other hand, auxin transport depends on the organization and dynamics of microfilaments (Zhu and Geisler, 2015). Auxin regulates both expansion and polarity of individual cells, as well as initiation and patterning of organs. Transient auxin concentration gradients underlie developmental processes such as meristem initiation, organ primordia formation, embryo morphogenesis, lateral root formation, as well as regulation of phyllotaxy and vascular tissue differentiation, photo- and gravitropic responses (Berleth and Sachs, 2001; Vanneste and Friml, 2009; Cardarelli and Cecchetti, 2014; Adamowski and Friml, 2015). Transient auxin concentration gradients result from local biosynthesis and polar cell-to-cell transport of the hormone. PAT is mediated by specific auxin uptake permeases and efflux carrier proteins that localize to plasma membrane in an asymmetric manner. PIN-FORMED (PIN) and ATP-binding cassette transporters/*P*-glycoprotein (ABCB/PGP) families are principal auxin efflux carriers whereas members of Auxin-Resistant 1/LIKE-AUX1 (AUX1/LAX) family are major auxin uptake carriers (Okada et al., 1991; Bennett et al., 1996; Gälweiler et al., 1998; Luschnig et al., 1998; Marchant et al., 1999; Swarup et al., 2001; Geisler and Murphy, 2006; Krecek et al., 2009; Zažímalová et al., 2010; Swarup and Péret, 2012; Adamowski and Friml, 2015). The regulation of PAT depends mainly on the action of auxin efflux carriers of the PIN and ABCB/PGP families (Geisler and Murphy, 2006; Vanneste and Friml, 2009; Adamowski and Friml, 2015). Both auxin influx permease AUX1, as well as efflux carriers (PINs and ABCBs) cycle between the plasma membrane and endosomal compartments (Geldner et al., 2001; Kleine-Vehn et al., 2006, 2011; Cho et al., 2007; Kleine-Vehn and Friml, 2008; Titapiwatanakun et al., 2009; Swarup and Péret, 2012; Cho and Cho, 2013; Wang et al., 2013). The crosstalk between auxin and actin is confirmed by findings showing that both localization and recycling of auxin importers and exporters depends partially on AFs (Geldner et al., 2001; Kleine-Vehn et al., 2006; Dhonukshe et al., 2008; Wu et al., 2015; Zhu and Geisler, 2015). However, the exact role of the actin cytoskeleton in PAT is still unresolved. It has been suggested that interactions between auxin and actin are not only dose- and time-dependent, but also species- and organ-dependent (Zhu and Geisler, 2015).

Several recent findings have shown that alterations in the cytoskeleton polymerization status are also critical for triggering PCD in plants (Smertenko et al., 2003; Thomas et al., 2006; Keech et al., 2010; Breeze et al., 2011; Keech, 2011; Smertenko and Franklin-Tong, 2011; Chang et al., 2015). Senescence is the terminal phase in organ development that involves a programmed degradation of cellular components. The resulting degradation products of senescing tissues are reused to support the growth of newly forming organs like leaves, roots, tubers, shoots, flowers, fruits, and seeds (Himelblau and Amasino, 2001; Maillard et al., 2015). Leaf senescence, the most well studied type of organ senescence in *Arabidopsis*, is characterized by the elevated expression of the senescence-associated genes (*SAGs*), early auxin-responsive small auxin-up RNA genes (*SAURs*), loss of chlorophyll, degradation of organelles, autolysis distinguished by fragmentation of the tonoplast and subsequent removal of the cytoplasm (Lohman et al., 1994; Weaver et al., 1998; Lin and Wu, 2004; Zentgraf et al., 2004; Balazadeh et al., 2008; Hou et al., 2013; Watanabe et al., 2013; Woo et al., 2013; Ren and Gray, 2015; Kim et al., 2016). Many *SAGs* encode proteins that drive breakdown of cellular components, such as short-chain alcohol dehydrogenase SAG13, and cysteine protease SAG12 (Weaver et al., 1997; Buchanan-Wollaston et al., 2003; Lin and Wu, 2004; Zentgraf et al., 2004; Watanabe et al., 2013; Woo et al., 2013; Kim et al., 2016). It has been demonstrated that the *SAG12* is specifically induced by developmental senescence, and *SAG13* by a range of senescence-inducing stress-treatments such as detachment, hormonal treatment, darkness, drought, wounding and pathogen attack. Therefore, it is proposed that *SAG12* could be the marker for age-related developmental senescence, and *SAG13* for stress-induced senescence or general cell-death (Schippers et al., 2007).

Auxin involvement in senescence has been observed to a much longer than the role of cytoskeleton (Hodge and Sacher, 1975; Noodén and Noodén, 1985; Lim et al., 2010; Kim et al., 2011; Ren and Gray, 2015; Cha et al., 2016). Nevertheless, the precise functions of auxin in leaf senescence remain unclear due to controversial results reporting either negative or positive role of auxin in leaf senescence regulation (Lim et al., 2010; Kim et al., 2011; Hou et al., 2013; Jibran et al., 2013; Woo et al., 2013; Khan et al., 2014; Ren and Gray, 2015; Cha et al., 2016). However, it has been suggested that auxin may promote leaf senescence through the expression of *SAUR36* gene in *Arabidopsis* (Hou et al., 2013).

Although the auxin impact on secondary metabolism in plants is not well understood, numerous investigations point to the auxin’s role in modulating flavonoid biosynthesis (Buer and Muday, 2004; Besseau et al., 2007; Lewis et al., 2011; Kuhn et al., 2016). Flavonoids, in turn, are considered to be endogenous regulators of auxin efflux carriers, suggesting a crosstalk between auxin- and flavonoid-dependent processes (Murphy et al., 2000; Brown et al., 2001; Buer and Muday, 2004; Peer et al., 2004; Besseau et al., 2007; Peer and Murphy, 2007; Santelia et al., 2008; Zažímalová et al., 2010). Flavonoid biosynthesis produces a variety of distinct flavonoid subclasses, including anthocyanins, a group of pink, red, purple or blue pigments widely produced in plants (Harborne and Baxter, 1999). In *Arabidopsis*, anthocyanins accumulate in variable amounts in leaves and stems, depending on light intensity and nutrition (Holton and Cornish, 1995; Gou et al., 2011). Accumulation of anthocyanins and acceleration of senescence are also well documented under certain stress conditions including reduced nitrogen levels and high light intensity (Feild et al., 2001; Peng et al., 2008; Sekhon et al., 2012; Misyura et al., 2013).

Even though myosin and AFs act in concert, the potential role of myosins in auxin responses is starting to be revealed, but the one in senescence and cell death largely remains addressed. It has been reported previously that myosin 3KO roots show moderate unresponsiveness to exogenous auxin treatment, exhibiting partially insensitive vacuoles (Scheuring et al., 2016). Because both the AF architecture and overall actin dynamics are altered in myosin 3KO cells (Peremyslov et al., 2010; Ueda et al., 2010; Cai et al., 2014; Scheuring et al., 2016), it is likely that the mutant cells are less responsive to physiological and developmental stimuli such as auxin signaling. Very recent findings of Abu-Abied et al. (2018) showed that the altered root architecture of the 3KO plants was in correlation with the reduced auxin gradient, and partial loss of PIN1 polarization in the stele cells. These results provide first evidence that PAT, at least partially, could be myosin-mediated process in *Arabidopsis*.

In this study, we assessed the potential roles of class XI myosins in mediating auxin response and cell death during floral development and leaf senescence, respectively. We used well-characterized class XI myosin triple gene knockout mutant 3KO (Ojangu et al., 2012) as it has exhibited a prominent phenotype including stunted growth, partially impaired shoot development, and premature leaf yellowing, suggesting a possible connection between myosin function, auxin distribution and senescence signaling. Investigation of the genetically rescued 3KOR (Peremyslov et al., 2012) line confirmed that the observed defects have a myosin-dependent nature. Auxin-related processes in 3KO background were monitored through evaluating the activity of the auxin-responsive reporters DR5::GUS and IAA2::GUS, and comparing the expression patterns of the auxin efflux carrier PIN1::PIN1-GFP and of myosin XI-K:YFP in floral development. Senescence-related processes were analyzed by measuring the contents of chlorophylls and anthocyanins, and following the cell integrity, and patterning of AFs in senescent leaf cells. In addition, relative expression levels of auxin-responsive and senescence-related genes were evaluated. Collectively, our data imply that class XI myosins contribute significantly to auxin responses, stress-induced senescence, and cell death in *Arabidopsis*. At that, we provide first genetic evidences that actomyosin cytoskeleton mediates senescence-processes in *Arabidopsis*. Moreover, our results indicate that there is a mutual crosstalk between actomyosin cytoskeleton, auxin-regulated and senescence-dependent processes, and secondary metabolism.

## Materials and Methods

### Plant Material and Growth Conditions

*Arabidopsis thaliana* (ecotype Columbia-0) seeds of *xi1* (Salk_022140; At1g17580), *xi2* (Sail_632_D12; At5g43900), *xik* (Salk_067972; At5g20490) T-DNA mutant lines and PIN1::PIN1-GFP (ecotype Landsberg erecta, Ler) line (N23889) were obtained from the Nottingham *Arabidopsis* Stock Centre. Myosin mRNA levels of single mutant T-DNA lines were determined earlier by RT-qPCR (Talts et al., 2016). The myosin triple mutant line *xi1 xi2 xik* (3KO in this work) was published previously (Ojangu et al., 2012). PIN1::PIN1-GFP (Ler) and IAA2::GUS (Ler) lines were backcrossed four times to Columbia-0 (Columbia or Col in this work) prior to phenotypic analyses. The genetic background of the 3KO line transformed with the gene encoding YFP-tagged myosin XI-K, *xi1 xi2 xik XI-K:YFP* (3KOR in this work), was described earlier (Peremyslov et al., 2012). Reporter lines DR5::GUS (Col-0) and IAA2::GUS (Ler) were obtained from Malcolm Bennett’s lab, and seeds of the 35S::GFP-fABD2-GFP (Wang et al., 2008) line from Elison B. Blancaflor’s lab. The genes of DR5::GUS and IAA2::GUS auxin reporters, and the 35S::GFP-fABD2-GFP actin marker were introduced into the 3KO line by crossing the plant lines.

Vernalized seeds were held in water at 4°C for 1 day before sowing in the soil containing 50% (v/v) vermiculite. Plants were grown in growth chambers under 16 h light/8 h dark period at 22 ± 2°C and 60% of relative humidity. For the seedling analysis seeds were surface sterilized and grown on 0.5 × MS medium (Murashige and Skoog, 1962) supplemented with 1% sucrose in climate chambers as described above.

For 1-*N*-naphthylphthalamic acid (NPA) treatments, primary inflorescences were dipped twice (with 3-days interval) with 100 μm NPA (Sigma-Aldrich) and 0.01% (v/v) Silwet L-77 as adapted from Nemhauser et al. (2000). NPA was dissolved in dimethyl sulfoxide (DMSO), and mock treatments were performed with Milli-Q water containing 0.1% (v/v) DMSO and 0.01% Silwet L-77. For latrunculin B (LatB) (Abcam) treatments, 50 mg of 7-day-old seedlings grown on agar plates were incubated for 6 h in 5 ml of liquid MS medium supplemented with 0.5 μM LatB (dissolved in DMSO), and mock treatments were performed with liquid MS medium containing 0.025% (v/v) DMSO.

### Quantitative Analysis of β-Glucuronidase (GUS) Activity in Plant Extracts

The activity of β-glucuronidase (GUS) can be determined in extracts of plant tissue using 4-methylumbelliferyl β-D-glucuronide (4-MUG) as a substrate. The 4-MUG fluorometric assay of plant extracts was performed to measure the GUS activity under the control of the DR5 promoter. For vegetative growth phase analysis 100 mg of 12-day-old seedlings and 23-day-old rosettes were collected and frozen. For generative growth phase analysis, 100 mg of primary inflorescence stems were collected when in height of 10–14 cm (floral transition stage) and 20–25 cm (silique formation stage). Protocol for quantitative GUS activity assay was adapted from Lewis and Muday (2009). Plant extract preparation was as follows: 150 μl GUS extraction buffer (50 mM sodium phosphate buffer pH 7.0; 10 mM EDTA pH 8.0; 0.1% SDS; 0.1% Triton X-100; 10 mM β-mercaptoethanol; 25 μg/μl PMSF) was added to the 100 mg of frozen tissues, homogenized at 28 Hz/min for 2 min (Qiagen TissueLyser), cell debris was removed by centrifugation (15 min 13,200 rpm at 4°C). The reaction was carried out by adding 50 μl of plant extract to the 450 μl pre-warmed reaction mixture (GUS extraction buffer supplemented with 2 mM 4-MUG), incubated 20 h at 37°C in darkness. The reaction was stopped by adding 100 μl of reaction mixture to 900 μl of ice-cold 0.2 M Na_2_CO_3_. 255 μl of the stopped reaction was loaded to the black 90-well microassay plates (Greiner) and protected from light. Released 4-methylumbelliferone (4-MU) (AppliChem) was excited at 340 nm and emission was measured at 492 nm using Tecan GENios Pro. Total protein content of extracts was measured following BioRad QuickStart 1 × Bradford protocol. Two to three experiments were performed with each tissue type, including 6–12 biological and two technical replicates per experiment. The GUS activity was expressed as picomoles of 4-MU produced per minute per milligram protein (pmol/min/mg).

### Microscopy

The expression pattern analysis of Columbia and 3KO plants stably expressing PIN1::PIN1-GFP, XI-K:YFP or 35S::GFP-ABD2-GFP was performed with Carl Zeiss LSM 510 META confocal laser scanning microscope. GFP or YFP was excited at 488 nm with argon laser and fluorescence was detected with 505–550 nm band-pass filter; band-pass filter 575–615 IR was used for chlorophyll autofluorescence detection. Dissected flower parts, buds, and floral primordia were immersed in 50% glycerol or 95% perfluorodecalin (Sigma-Aldrich). Silicon spacer was applied in between glass slide and cover slip to prevent crushing tissues. Presented images show *Z*-stacks of confocal images combined into single image by maximal intensity projections using Zeiss LSM Image Browser software.

For histochemical analysis, DR5::GUS and IAA2::GUS plants were treated according to a standard protocol (Weigel and Glazebrook, 2002), immersed in Mowiol mounting medium, and analyzed under a light microscope. Images of DR5::GUS plants and trypan blue stained leaves were captured with digital camera Nikon D800E using a slide copying adapter ES-1. Images of IAA2::GUS inflorescences and flowers were captured with Zeiss SteREO Discovery.V8.

Scanning electron microscope (SEM) analysis was performed with Carl Zeiss EVO LS15. For flower architecture analysis in *Arabidopsis*, flower parts were dissected on double-sided tape using fine needle (27G) and SEM images of unfixed and uncoated flower tissues were captured using a reduced vacuum mode (100–200 Pa).

Adobe Photoshop CS6 was used to assemble photographs, indicate details, and measure the intensity of trypan blue staining on leaf photographs (as mean value of the blue channel). ImageJ2 software was used to analyze the PIN1-GFP fluorescence in single optical longitudinal sections (0.71 μm thick) of outer epidermal cells of stage 8 gynoecia. GFP fluorescence intensities of apical and lateral membranes of each cell was measured as quotients: mean gray values divided by values of selected areas. The ratios of the GFP fluorescence of apical membranes versus lateral membranes of each cell were used to evaluate the distribution of PIN1-GFP: the average ratio value of Columbia cells (3.3) was set as threshold to discriminate the cells with less polarized (<3.3) and more polarized (>3.3) PIN1-GFP distribution. The lower the ratio number was the less the fluorescence between apical and lateral membranes differentiated.

### Quantification of Total Chlorophyll Content

For chlorophyll extraction, the fifth and sixth leaves from 23-day-old rosettes (before bolt formation) were dissected and frozen immediately. For dark-induced senescence, dissected leaves were placed in 30 ml of Milli-Q water and incubated for 3 days in growth chamber at darkness. One biological replicate contained six leaves – the fifth and sixth leaves from three different 23-day-old rosettes. Three independent experiments with six biological replicas in each were performed. Chlorophyll was extracted from the rosette leaves before and after dark-treatment as described previously (Hu et al., 2013; Misyura et al., 2013). Briefly, 1 ml of ice cold 80% acetone in 0.2 M Tris-buffer (pH 8.0) was added to frozen samples, homogenized with chrome-steal beads in TissueLyser 20 Hz/min for 1 min, and incubated 12 h in the dark at 4°C. Next day, samples were centrifuged 15 min 3000 rpm at 4°C. The extraction with 80% acetone solution was repeated three times, supernatants of every extraction were collected. 1 ml of extract was transferred to a disposable polymethyl methacrylate (PMMA) cuvette, and absorbance at 645 and 663 nm were measured. The total chlorophyll concentration in fresh weight (mg/g) were calculated using the equation:

$$C\text{hlorophyll a + b=}\frac{[(8.05\times {OD}_{663})+\left(8.05\times {OD}_{645}\right)]\times V\left[\text{extract volume}\left(ml\right)\right]}{1000\times W\left[\text{fresh weight}\;\left(g\right)\right]}$$

### Trypan Blue Staining

For trypan blue staining the fifth leaves from 21-day-old rosettes were selected. Fresh or dark treated leaves were boiled in lactophenol (10 ml of lactic acid, 10 ml of glycerol, 10 ml of liquid phenol, and 10 ml of distilled H_2_O) containing 10 mg of trypan blue for 1 min. Tissues were cleared in alcoholic lactophenol (2:1 95% ethanol:lactophenol) for 2 min, washed in 50% ethanol at room temperature, and stored in Milli-Q water. For the analysis, leaves were immersed in Mowiol mounting medium.

### Quantification of Anthocyanin Content

Anthocyanin extraction from *Arabidopsis* seedlings was performed as described previously (Nakata and Ohme-Takagi, 2014). In brief, 100 mg of 7-day-old seedlings were frozen in liquid nitrogen, homogenized at 28 Hz/min for 2 min (TissueLyser), and suspended into five volumes of extraction buffer (45% methanol and 5% acetic acid). Cell debris was removed by centrifugation (20 min 13,200 rpm at room temperature). The relative anthocyanin content was calculated from the absorbance at 530 and 657 nm (Shimadzu Biospec Mini) using the equation:

$$\text{Relative anthocyanin content = }\frac{[({OD}_{530}-\left(0.25\times {OD}_{657}\right)]\times V\left[\text{extract volume}\left(ml\right)\right]}{1000\times W\left[\text{fresh weight}\;\left(g\right)\right]}$$

### RNA Extraction and Reverse Transcription – Quantitative Real-Time PCR (RT-qPCR)

Total RNA was extracted from 50 mg of plant material according to the method described by Oñate-Sánchez and Vicente-Carbajosa (2008). Buffer volumes were scaled up three times. Expression levels of *AUX1*, *IAA2*, *PIN1*, *PIN3*, *PIN4*, *PIN7*, *SAUR36*, *SAG12*, and *SAG13* genes were analyzed. RNA was extracted from 7-day-old seedlings, 21-day-old leaves before and after dark-induced senescence, and mature inflorescences. cDNA was synthesized from 5 μg of DNase-treated RNA using Maxima Reverse Transcriptase (Thermo Scientific) and random hexamer primer. cDNAs were diluted twofold for qPCR. All RT-qPCR reactions were performed in 384-well plates on the LightCycler 480 instrument (Roche Applied Science). qPCR reactions were performed in duplicate and *C*q values were averaged. Each 7 μl reaction contained 1.4 μl 5x HOT FIREPol^®^ EvaGreen^®^ qPCR Mix Plus (no ROX) (Solis Biodyne), 0.7 μl diluted cDNA and 3.5 pmol of each primer. qPCR conditions were as follows: initial denaturation at 95°C for 12 min, followed by 45 cycles of 95°C for 15 s, 59°C for 30 s and 72°C for 30 s. All primers used for qPCR experiments were designed for an annealing temperature of 60–62°C. Primers used for qPCR experiments are listed in Supplementary Table S1. Primers for reference genes were chosen as described previously (Czechowski et al., 2005; Supplementary Table S1). In all experiments, three reference genes were used for normalization: SAND, UBC, and expressed sequence EX70. Five to six biological replicates were analyzed in each experiment. Reference gene stability was analyzed using GeNorm M and coefficient of variation (CV) in qbase^PLUS^ software (Hellemans et al., 2007). Statistical analysis (One-Way ANOVA) was performed with qbase^PLUS^ software.

## Results

### The Activity of the Auxin-Responsive Reporters Is Reduced in 3KO Plants

Auxin signaling regulates all aspects of plant development, including determination of apical dominance during shoot growth, through finely tuned concentration gradients (Berleth and Sachs, 2001; Vernoux et al., 2010). Investigating the overall plant morphology, we noticed that the shoot development of 3KO plants varied significantly as mutant plants frequently displayed partially reduced apical dominance, and increased formation of axillary branches (Supplementary Figure S1). The average number of secondary inflorescences per cm of primary stems in 3KO plants was 1.8-fold, and the one of tertiary inflorescences 2.4-fold higher in comparison with Columbia control (Supplementary Figure S1). To assess if the developmental defects of 3KO plants have auxin-dependent nature, we used the auxin-responsive promoter-reporters DR5::GUS (β-glucuronidase), and IAA2::GUS which have been widely used as relevant markers to study endogenous auxin responses (Ulmasov et al., 1997; Shibasaki et al., 2009).

**FIGURE 1:**
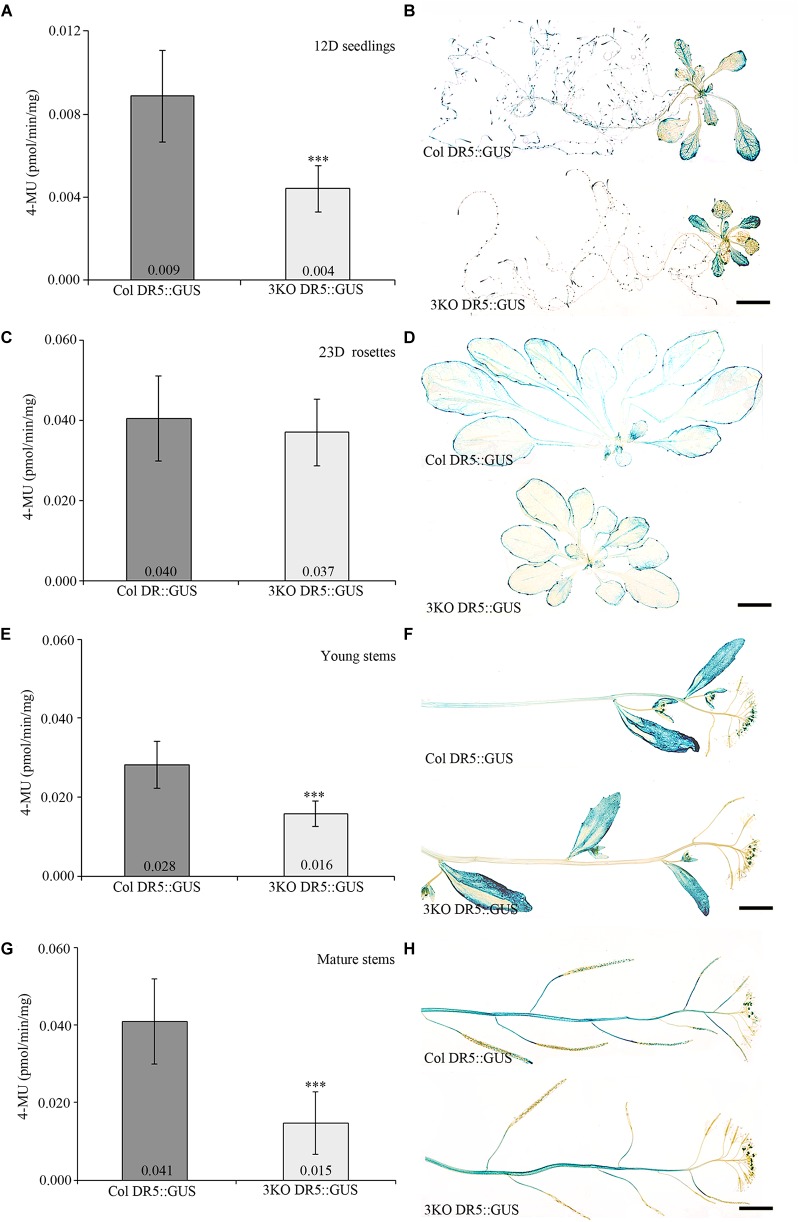
Quantitative and histochemical detection of GUS activity driven by the DR5 promoter. **(A,B)** 12-day-old (12D) seedlings, *n* = 27–29; **(C,D)** 23-day-old (23D) rosettes, *n* = 13–17; **(E,F)** young primary inflorescence stems, *n* = 21–27; **(G,H)** mature primary inflorescence stems with siliques, *n* = 36–48. Data represent average values; error bars represent SD; ^∗∗∗^*p* < 0.001 (Student’s *t*-test). Scale bars are 5 mm.

Fluorescent GUS assay using 4-methylumbelliferyl-β-D-galactopyranoside (4-MUG) fluorophore was used to measure DR5 promoter activity in plant extracts. Extracts prepared from 3KO seedlings stably expressing DR5::GUS (3KO DR5::GUS) showed 2.3-fold lower DR5 promoter activity when compared to the Columbia wild type control (Col DR5::GUS; Figure 1A). More specifically, histochemical GUS staining revealed reduced coloration in the roots of 3KO DR5::GUS plants (Figure 1B). GUS activity in rosette leaves of 3KO DR5::GUS plants was comparable with Columbia control (Figures 1C,D). Young 10–14 cm inflorescence stems and mature 20–25 cm inflorescence stems of the 3KO DR5::GUS plants showed 1.8-fold (Figure 1E) and 2.7-fold decrease (Figure 1G) in GUS activity, respectively, in comparison with Columbia control. Histochemical GUS assays revealed reduced GUS activity in the stems (Figure 1F) and siliques (Figure 1H) of 3KO DR5::GUS plants.

**FIGURE 2:**
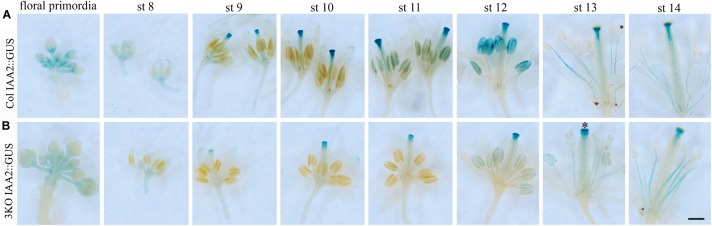
Histochemical detection of GUS activity driven by the IAA2 promoter. Inflorescences from young primary stems of Col IAA2::GUS **(A)**, and 3KO IAA2::GUS **(B)** lines were used for histochemical staining. Flowers from one inflorescence are displayed in developmental sequence: bunch of floral primordia, pre-anthesis stages 8–12 (st 8–12), the first opened flower at anthesis (st 13), and the second opened flower at post-anthesis (st 14). **(B)** In 3KO plants, the GUS staining is weaker in stigmas, styles and transmitting tracts of pistils at stages 9–13 (st 9–13) and anthers at stages 11–12 (st 11–12) in comparison with Col. Black star indicates GUS staining of 3KO stigmas at anthesis (st 13), indicating that the development of mutant stigmas delays. Scale bar is 400 μm.

The IAA2::GUS reporter was used both for quantitative and histochemical analysis. Quantitative analysis of the IAA2::GUS activity in Columbia and 3KO extracts showed the similar reduction as the DR5::GUS reporter (data not shown). Histochemical staining of young inflorescences of Columbia and 3KO plants stably expressing IAA2::GUS showed similar staining patterns but different staining intensities (Figure 2). Throughout the flower development, the GUS staining in 3KO IAA2::GUS pistils (stigma, style and transmitting tract) (Figure 2B; st 9–13) and anthers (Figure 2B; st 10–11) was weaker than that in Columbia IAA2::GUS line. In addition, the GUS staining of 3KO stigmas at anthesis (Figure 2B; st 13) highlighted the retarded development of 3KO stigmas which was demonstrated previously (Ojangu et al., 2012).

We also analyzed the effect of NPA, the polar auxin transport inhibitor, on the IAA2::GUS expression. For this, inflorescences of Columbia IAA2::GUS and 3KO IAA2::GUS plants were dipped twice with 100 μM NPA. The histochemical examination of primary inflorescences revealed that NPA-treatment led to increased IAA2::GUS activity both in Columbia and 3KO inflorescences, when compared to DMSO-treated controls (Supplementary Figure S2). However, the responses to NPA were somewhat different in Columbia and 3KO. In NPA-treated Columbia, the strong GUS staining was spread all over the gynoecia, but it did not accumulate in valves of 3KO IAA2::GUS gynoecia (Supplementary Figure S2). Slightly weaker staining of pedicles, petals and sepals was noticeable in 3KO IAA2::GUS also (Supplementary Figure S2).

These results show that the simultaneous inactivation of three class XI myosins reduces the auxin responses of different *Arabidopsis* tissues.

### Stable Expression of PIN1-GFP in 3KO Enhances Abnormalities in Flower Formation and Growth

In *Arabidopsis*, developmentally important auxin gradients are generated during PAT by modulating the organization and dynamics of actin cytoskeleton (Geldner et al., 2001; Ivakov and Persson, 2013; Zhu and Geisler, 2015). As the aberrant shoot development in 3KO plants correlated with the reduced auxin response, we further investigated if this was due to possible deviations in the distribution of auxin efflux carrier PIN1, since it plays an essential role in flower and inflorescence formation (Okada et al., 1991). For this, we examined the effect of stable expression of PIN1::PIN1-GFP in 3KO (3KO PIN1-GFP) and Columbia control plants (Col PIN1-GFP).

**FIGURE 3:**
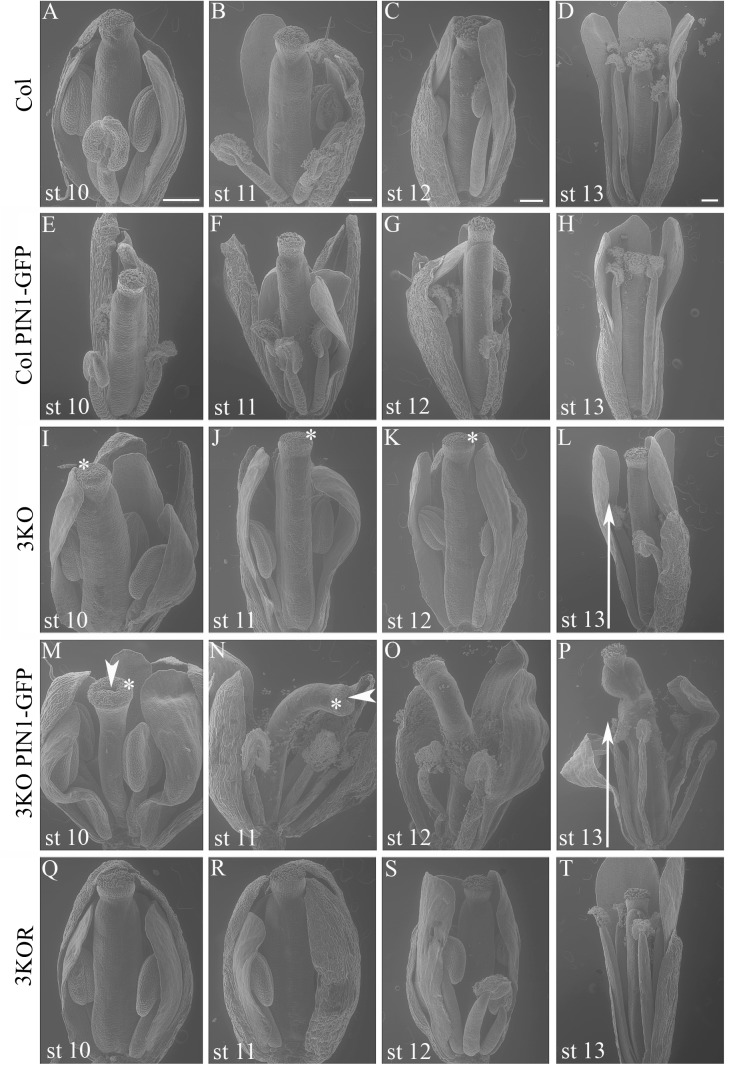
Flower architecture during anthesis and pre-anthesis development. Flowers of primary inflorescences of Col **(A–D)**, Col PIN1-GFP **(E–H)**, 3KO **(I–L)**, 3KO PIN1-GFP **(M–P)**, and 3KOR **(Q–T)** plants were selected for assessment. Flowers from one inflorescence were picked sequentially beginning with the first opened flower at anthesis (st 13), and following with three pre-anthesis buds at stages 12, 11, 10 (st 12, st 11, and st 10). Developmental deviations of 3KO **(I–L)** and 3KO PIN1-GFP **(M–P)** flowers are indicated as following: white arrows point to the retarded elongation of anther filaments during anthesis **(L,P)**; asterisks mark retarded elongation of stigmatic papillae **(I–K,M,N)**. Notice also deformed architecture of 3KO PIN1-GFP pistils: bent gynoecia **(M–P)**, valveless pistil with swollen stigma **(M)**, and bent sepals **(M,O,P)**; white arrowheads indicate delayed apical closure of gynoecia **(M,N)**. Notice that the normal flower development is recovered in complemented line 3KOR **(Q–T)**. Scale bars are 200 μm.

It is previously described that in 3KO plants, the fertility is slightly decreased as the pistil maturation (elongation of stigmas) partially delays (Ojangu et al., 2012). Interestingly, when examining the flowers at anthesis (stage 13/14) by using SEM, we found that the elongation of stamen filaments in parental 3KO line frequently delayed also (Figure 3L; white arrow). This indicated that during flower anthesis of the 3KO plants the rapid growth spurt of stamen filaments is partially retarded, and thus stamens often do not reach stigmas in time for proper pollination.

**FIGURE 4:**
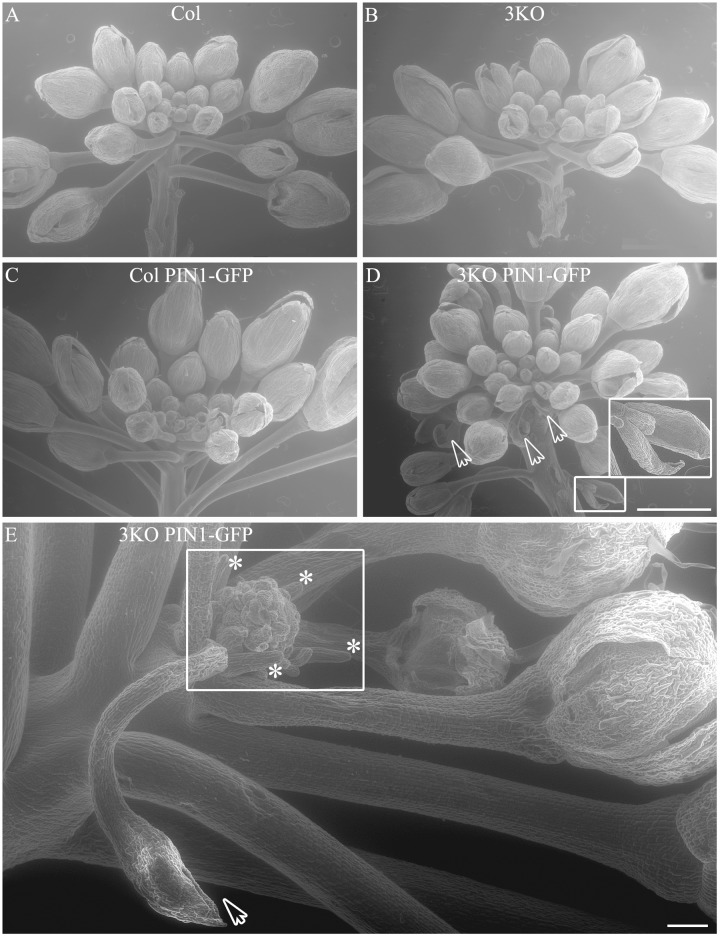
Architecture of primary infloresence apices. Primary inflorescence apices of Col **(A)**, 3KO **(B)**, Col PIN1-GFP **(C)** and 3KO PIN-GFP **(D,E)** plants. Notice that the development of 3KO PIN1-GFP primary inflorescences is irregular **(D,E)**. **(D)** Inflorescence apices of 3KO PIN-GFP primary stem contain occasionally more floral primordias as the bunch of flower buds is denser in comparison with Col, 3KO, or Col PIN1-GFP. White framed magnification and white arrows indicate undeveloped flower buds, empty bud-like structures. Scale bar is 1 mm. **(E)** Inflorescence apices of 3KO PIN-GFP primary shoots contain occasionally less floral primordias as the bunch of flower buds is sparser in comparison with Col, 3KO, or Col PIN1-GFP. White frame indicates the loss of meristem identity as newly formed floral primordia look severly deformed, asterisks indicate pin-like structure of these primordia. White arrow indicates empty bud-like structure. Scale bar is 100 μm.

Even more surprising was the finding that previously described semi-sterile phenotype of 3KO plants was exacerbated in the 3KO PIN1-GFP line. SEM imaging revealed that the architecture of inflorescences, formation of floral primordia (Figure 4), and development of floral organs (Figure 3) were significantly affected in 3KO PIN1-GFP plants. The 3KO PIN1-GFP inflorescences displayed very irregular architecture being occasionally denser (Figure 4D and Supplementary Figure S3) or sparser (Figure 4E) in comparison with Columbia, 3KO or Col PIN1-GFP plants, indicating that the inflorescence meristem was partially disturbed. No matter if meristem produced more or less floral primordia (Figures 4D,E) their capability to develop into normal flowers was partially impaired. Some flowers showed completely arrested development (Figures 4D,E) and others exhibited a range of morphologies, from near-normal to severely deformed (Figure 3 and Supplementary Figure S3). Undeveloped flowers did not contain pistils, petals or anthers, and consisted only of one to three sepal-like structures (Figures 4D,E; white arrows). In extreme cases, the inflorescence meristem of the 3KO PIN1-GFP line produced only ∼20 near-normal flowers or floral buds. Thereafter, the emergence of new flowers stopped, since only needle-like floral primordia were formed (Figure 4E; asterisks). However, the most prevalent flower deformation was a significantly bent shape of pistils (Figures 3N–P). Delayed apical closure of gynoecia (Figures 3M,N; white arrowheads) and pistils with decreased valves, and swollen style and stigma region (Figure 3M) were frequently observable during late stages of 3KO PIN1-GFP flower development. Besides serious aberrations, the flowers of 3KO PIN1-GFP plants exhibited also same deviations as parental 3KO line: retarded elongation of stamen filaments (anthesis stage 13/14) (Figure 3P; white arrow) and stigmatic papillae (pre-anthesis stage 11) (Figure 3N; asterisk). Modified architecture of 3KO PIN1-GFP inflorescences and flowers indicated that even the modest overexpression of PIN1 under native promoter may disturb auxin responses, and thus affect developmental decisions, when the actomyosin cytoskeleton is simultaneously affected.

### Partial Loss of PIN1-GFP Polarization During 3KO Flower Development

**FIGURE 5:**
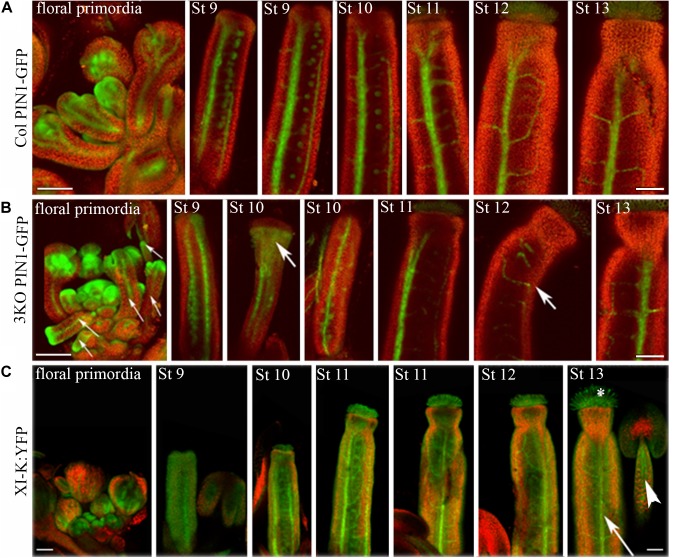
Expression patterns of PIN1::PIN1-GFP and XI-K:YFP throughout flower development. **(A)** In Col control, the PIN1-GFP is expressed in septums or valve margins of pistils and in floral primordia. **(B)** In 3KO, a prevalent tissue pattern of PIN1-GFP is similar to control with some exceptions: the loss of PIN1 polarization is visible in valveless pistil (white bold arrow, St 10), and pin-like floral primordia are indicated with white thin arrows. White bold arrows indicate the deformed shapes of pistils (St 10, St 12). **(C)** In 3KOR, the XI-K:YFP fluorescence is visible in anther filaments (white arrowhead), stigmatic papillae (white asterisk), septum or valve margins (long white arrow), and floral primordia. St 9–13 indicate developmental stages of pistils. The red signal represents chlorophyll autofluorescence. Scale bars are 100 μm.

Next, the distribution of auxin efflux carrier PIN1 in flower tissues of 3KO and Columbia plants was visualized with expression of the PIN1::PIN1-GFP. Examination of the PIN1-GFP distribution in flower tissues of Columbia plants revealed its presence in septum or valve margins of pistils, as well as in floral primordia (Figure 5A). The PIN1 patterning in the Columbia gynoecia was consistent with previously published data (Zúñiga-Mayo et al., 2014). The overall patterning of the PIN1-GFP in 3KO floral tissues was broadly the same with some exceptions. For instance, in heavily deformed 3KO pistils, such as valveless gynoecia, the PIN1-GFP signal was spread all over the gynoecium (Figure 5B; St 10; white bold arrow). Similarly, PIN-GFP pattern in developing 3KO floral primordia was occasionally aberrant (Supplementary Figure S4).

**FIGURE 6:**
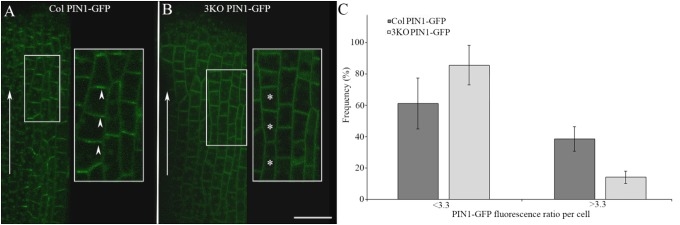
PIN1-GFP localization in epidermal cells of developing gynoecia. **(A,B)** PIN1-GFP localization in an longitudinal optical section of the outer epidermal cells of stage 8 gynoecia. **(A)** Col PIN1-GFP epidermal cells (white framed magnification) show polarized distribution of PIN1; white arrowheads exemplify the preferred localization of PIN1 at apical membranes. **(B)** 3KO PIN1-GFP epidermal cells (white framed magnification) show partial loss of PIN1 polarization; white asterisks exemplify the unpolarized distribution of PIN1 both in apical and lateral membranes. White arrows indicate the auxin flow from base to apex. Large images are projected from a stack of 9–12 optical slices at a 0.71 μm interval and white framed magnifications indicate projections of three optical slices. Scale bar is 20 μm. **(C)** Histogram shows frequency of cells with different PIN1-GFP polarization values. The PIN1-GFP polarization of each cell was evaluated as the ratio of apical membrane fluorescence to lateral membrane fluorescence. The ratio value of 3.3 was set as threshold to discriminate the cells with less polarized (<3.3) and more polarized (>3.3) PIN1-GFP distribution. Error bars represent SD.

We examined the PIN1-GFP polarization in developing gynoecia at the cellular level because the significant pistil deformation was the most prominent phenotype of the 3KO PIN1::PIN1-GFP line. PIN1-GFP localization in longitudinal optical sections of the outer epidermal cell layer of stage 8 gynoecia was evaluated as ratio of apical membrane fluorescence to lateral membrane fluorescence of each cell. In outer epidermal cell layer of Columbia gynoecia, the PIN1-GFP was prominently apically localized (Figure 6A; arrowheads), the average ratio of GFP fluorescence of apical membranes versus lateral membranes was 3.3 ± 1.75 (*n* = 31). Epidermal cells of 3KO gynoecia displayed comparable PIN1-GFP fluorescence on both apical and lateral membranes (Figure 6B; asterisks), the average ratio of GFP fluorescence of apical membranes versus lateral membranes was 2.3 ± 1.33 [*n* = 42; *p* < 0.05 (Student’s *t*-test)]. To further validate the frequency of less and more polarized PIN1-GFP distribution of epidermal cells in Columbia and 3KO gynoecia, the ratio value of 3.3 of the GFP fluorescence was set as threshold (Figure 6C). The quantification showed that the ratio values less than 3.3 were observed in 86% of epidermal cells of developing 3KO PIN1-GFP gynoecia (Figure 6C) while in Col PIN1-GFP gynoecia, the frequency was 61% (Figure 6C). The ratio values higher than 3.3 were observed only in 14% of 3KO PIN1-GFP cells but in 39% of Col PIN1-GFP cells (Figure 6C). The gynoecium is the last organ to initiate from the floral meristem (Larsson et al., 2014), and apical domains such as the style and stigma are last structures which emerge during gynoecium development. The partial loss of PIN1 polarization in 3KO PIN1-GFP line is in accordance with severe pistil defects and indicated that the apical domains (style and stigma) of the developing gynoecium may not be sufficiently supplied with auxin in 3KO PIN1-GFP background. These results imply that class XI myosins, at least partially, contribute to the localization of PIN1 during floral development.

### Myosin XI-K Is Expressed Throughout Floral Development

Despite the fact that class XI myosins share functional redundancy, it is known that the myosin XI-K plays important roles in such processes as membrane trafficking, cell expansion and division, plant growth, and fertility (Ojangu et al., 2007, 2012; Peremyslov et al., 2008, 2010, 2012; Avisar et al., 2012; Park and Nebenführ, 2013; Abu-Abied et al., 2018). Accordingly, to validate the XI-K role in flower development, we performed SEM analysis of the genetically rescued 3KOR line (Peremyslov et al., 2012), and found that the normal inflorescence development, including the elongation of stigmas and anther filaments, was restored in this plant line (Figures 3Q–T). Further, XI-K:YFP fluorescent signal in floral primordia, pistils and stamen filaments was examined (Figure 5C). The expression patterns of XI-K:YFP and PIN1::PIN1-GFP partially overlapped in the floral primordia, as well as in septum and valve margins of developing pistils (Figure 5C). At the same time, the XI-K:YFP showed distinct expression patterns in stigmas and anther filaments (Figure 5C), in comparison with PIN-GFP (Figures 5A,B). Both the expression pattern analysis, and flower architecture evaluation of the 3KOR line indicate that myosin XI-K contributes to the growth of floral organs, and thus to fertility.

### The Senescence and Cell Death of 3KO Leaves Is Accelerated

**FIGURE 7:**
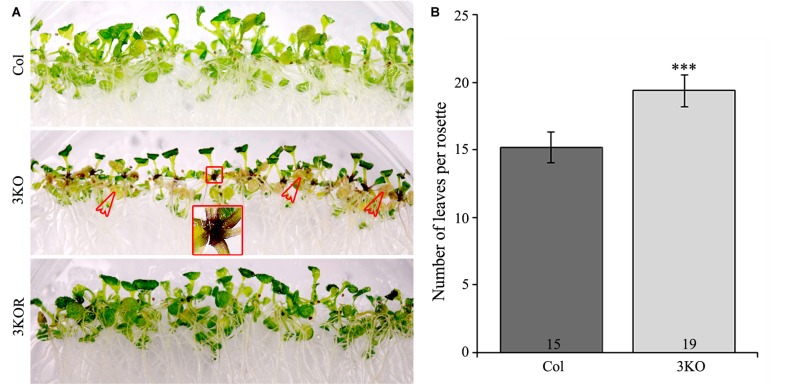
Premature leaf senescence of seedlings and number of rosette leaves. **(A)** The accelerated yellowing of cotyledons of 12-day-old 3KO seedlings is indicated with red empty arrows. Red framed magnification shows the dark purple coloration of root-shoot junction of the 3KO seedlings. **(B)** Number of rosette leaves of 23-day-old Col and 3KO plants. Data represent average values; error bars represent SD; *n* = 21–23; ^∗∗∗^*p* < 0.001 (Student’s *t*-test).

Despite the delayed bolt formation and extended lifespan, described earlier (Peremyslov et al., 2010; Ojangu et al., 2012), we found that the 3KO plants displayed premature senescence of rosette leaves. Physical signs of early aging were particularly striking in the 12-day-old 3KO seedlings, which showed significant yellowing of cotyledons in comparison with Columbia or 3KOR line (Figure 7A). At the same time, 23-day-old mature rosettes (at bolt formation) of the 3KO plants produced 27% more leaves than those of Columbia control (Figure 7B). Given the very limited data on contributions of cytoskeleton to senescence and cell death in plant cells (Smertenko et al., 2003; Keech et al., 2010; Keech, 2011; Smertenko and Franklin-Tong, 2011), these observations prompted us to further explore myosin’s role in these processes.

**FIGURE 8:**
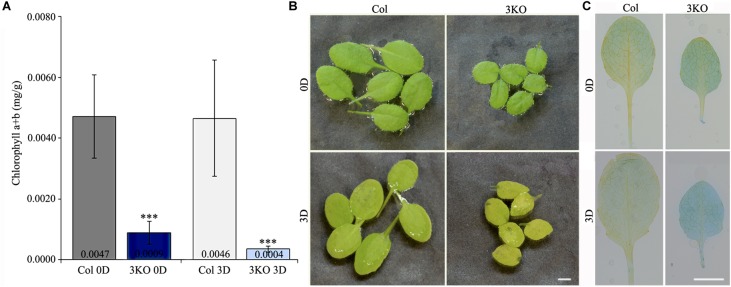
Senescence of rosette leaves. **(A)** Chlorophyll content of 21-day-old detached rosette leaves (fifth and sixth) before (0D) and after (3D) dark induced senescence. **(B)** Photographs of 21-day-old detached rosette leaves before (0D) and after (3D) dark induced senescence. Significant yellowing of 3KO leaves is visible after the dark treatment (3D). **(C)** Trypan blue staining of 21-day-old detached rosette leaves (fifth and sixth) before (0D) and after (3D) dark induced senescence. Significant blue staining of 3KO leaves is visible after the dark treatment (3D). Data represent average values; error bars represent SD; *n* = 15–16; ^∗∗∗^*p* < 0.001 (Student’s *t*-test). Scale bars are 4 mm.

First, to assess the senescence of 3KO plants, we measured chlorophyll content of detached rosette leaves before and after dark-induced senescence. Chlorophyll was extracted from the fifth and sixth leaves of 21-day-old rosettes. Quantification showed that the chlorophyll content in 3KO leaves before dark-treatment (Figure 8A; 3KO 0D) was only 19% of that in Columbia. After 3 days in darkness, the chlorophyll content of 3KO leaves was 9% of that of Columbia (Figure 8A; 3KO 3D). Photographs of detached leaves also show significant yellowing of the 3KO leaves after dark-treatment (Figure 8B; 3KO 3D).

Second, we employed trypan blue staining to distinguish the extent of cell death in rosette leaves before and after dark incubation (Figure 8C). Before dark incubation, at day 21 of rosette growth, the fifth leaves of both WT and 3KO plants were weakly stained with trypan blue (Figure 8C; Col 0D, 3KO 0D). Measurement of blue channel intensity of leaf pictures showed that after dark incubation, the trypan blue staining of Columbia leaves was 1.6 times, and the one of 3KO leaves 2.1 times more intense in comparison with untreated Columbia (Figure 8C; Col 3D, 3KO 3D), indicating the prevalence of dying cells in mutant leaves. In addition, the measurement of total protein content revealed that after dark-treatment, the total protein concentration of 3KO leaves was 2.8-fold lower, and the one of Columbia was 1.6-fold lower in comparison with untreated controls (3KO 0D, Col 0D), respectively (Supplementary Figure S5).

**FIGURE 9:**
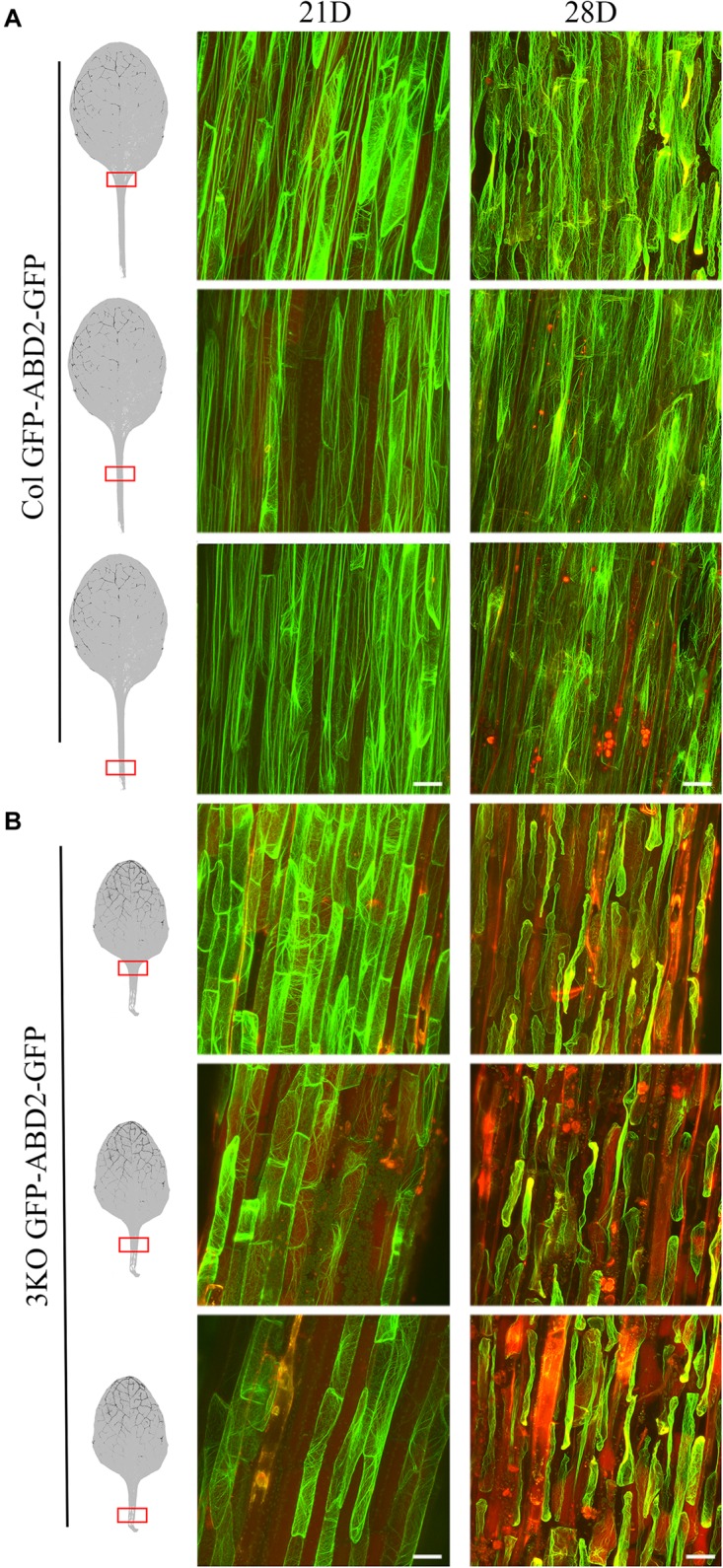
Cell integrity and architecture of actin arrays in abaxial epidermal cells of petioles of the fifth rosette leaf. Three petiole areas (near the leaf blade, in the middle, and near the proximal end) were examined as indicated with red boxes on leaf images. **(A)** Actin arrays in epidermal cells of Col petioles at days 21 (21D) and 28 (28D) of rosette growth. At 28 days, Col petioles show some loss of cell integrity in area near the leaf blade. **(B)** Actin arrays in epidermal cells of 3KO petioles at days 21 (21D) and 28 (28D) of rosette growth. Epidermal cells of 3KO petioles show prominently transverse actin arrays at day 21 when compared to the Col. 3KO petiole at 28 days post-sowing show significant plasmolysis of epidermal cells in comparison to Col. The red signal represents chlorophyll autofluorescence. Images are projected from a stack of 22–55 optical slices. Scale bars are 50 μm.

Third, to monitor the cell integrity and architecture of actin arrays in abaxial epidermal cells of the fifth rosette leaf’s petiole, we used stable expression of an AF tracer GFP-ABD2-GFP under control of the 35S promoter (Figure 9). Epidermal cells of leaf petioles were selected for examination as the cell growth, and AF organization defects of 3KO plants are most pronounced in longest cells, such as root hairs and petiole cells, as demonstrated by Peremyslov et al. (2010). Since 3KO leaf petioles are 50% shorter than those of Columbia we selected three areas of leaf petioles for examination: near the leaf blade, in the middle, and near the proximal end (Figure 9). The cell integrity and the organization of the AFs was compared between leaves of 21- and 28-day-old rosettes of 3KO and Columbia plants. At day 21 of rosette growth, the petiole cells were intact, and the GFP-ABD2-GFP decorated thick longitudinal cables in epidermal cells of Columbia leaf petioles (Figure 9A; Col 21D), and remarkably thin and prominently transverse filaments in 3KO cells (Figure 9B; 3KO 21D). At 28 days, GFP-ABD2-GFP labeled prominently longitudinal cables in Columbia cells (Figure 9A; Col 28D) and only traces of AFs in the 3KO petiole cells (Figure 9B; 3KO 28D). This was due to massive plasmolysis and cell shape deformation of 3KO petioles. In Columbia, less pronounced changes in AF organization and cell shape were found: 66% of petioles (*n* = 12) showed some plasmolysis at the area near the leaf blade, and only 25% of petioles showed massive plasmolysis (Figure 9A; 28D). This indicated that in 3KO plants, at day 28 of rosette growth, the cell death of older rosette leaves was significantly progressed in comparison with Columbia. Taken together, the results of decreased chlorophyll content, increased trypan blue staining, and premature plasmolysis in 3KO mutant show that the loss of integrity of actomyosin cytoskeleton induces premature senescence and thus cell death in *Arabidopsis* leaves.

### The Anthocyanin Pigments Accumulate in 3KO Tissues

**FIGURE 10:**
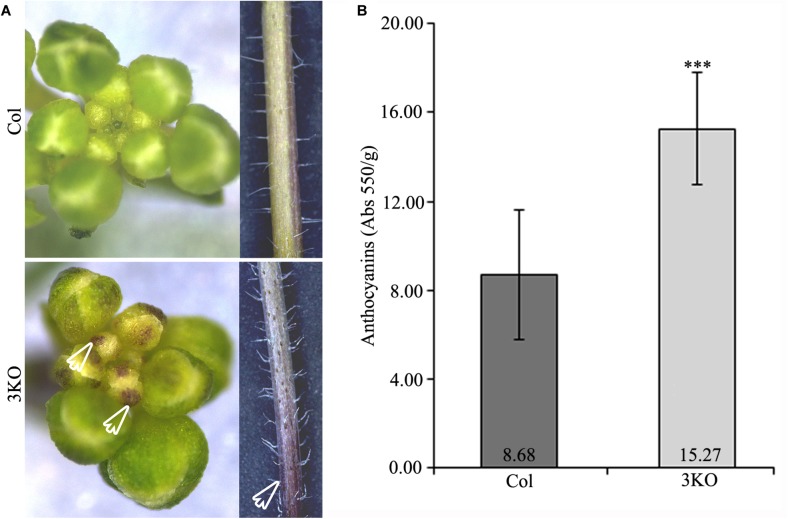
Purple pigmentation of primary inflorescence shoots and anthocyanin content. **(A)** White empty arrows indicate the purple pigmentation of floral buds and stem of the 3KO primary inflorescence axis. **(B)** Average anthocyanin content of 7-day-old seedlings. Data represent average values; error bars represent SD; *n* = 23–44; ^∗∗∗^*p* < 0.001 (Student’s *t*-test).

Eventually, when investigating the overall 3KO phenotype, we often observed an accumulation of purple pigments in various tissues including root-shoot junctions of seedlings (Figure 7A; red framed magnification), floral buds and basal parts of inflorescence stems (Figure 10A; white empty arrows). Such increased purple pigmentation of plant tissues is associated primarily with anthocyanin accumulation (Gou et al., 2011; Misyura et al., 2013; Mushtaq et al., 2016). To validate this assumption, the anthocyanin content of 7-day-old seedlings was determined spectrophotometrically (Figure 10B). It was found that anthocyanin content in the 3KO seedlings was 1.9-fold higher than that in Columbia control (Figure 10B). The anthocyanin accumulation rate in the 3KO rosettes and inflorescence stems was similar to that in seedlings (data not shown). This excessive anthocyanin accumulation is likely an accompanying effect related to reduced auxin response and accelerated senescence in the 3KO plants.

### Expression of the Auxin-Responsive and Senescence-Associated Genes Is Altered in 3KO

The main mechanism by which auxin- and senescence-responses are converted into cellular responses is via changes in transcription (Zentgraf et al., 2004; Paponov et al., 2008; Kim et al., 2016). To examine, if the elimination of multiple class XI myosins or the stable expression of *PIN1::PIN1-GFP* affect the regulation of the auxin-responsive and senescence-associated genes, we measured the relative expression levels of the selected mRNAs in seedlings, inflorescences, and rosette leaves of the Columbia, 3KO, 3KO PIN1-GFP, and Col PIN1-GFP plants using RT-qPCR. To assess auxin-related processes, we followed the expressions of auxin importer (*AUX1*) and exporter (*PIN1*, *PIN3*, *PIN4*, *PIN7*) genes whose expressions are related to flower development (Krecek et al., 2009; Lampugnani et al., 2013). We also measured the level of *IAA2* as its expression is closely related to endogenous auxin (Shibasaki et al., 2009). To distinguish stress- and age-induced senescence-related processes, we followed the relative expressions of *SAG13* and *SAG12* genes, respectively (Swartzberg et al., 2006; Schippers et al., 2007; Hou et al., 2013). To assess mutual influences between auxin- and senescence-related processes the level of auxin-responsive *SAUR36* was measured.

**FIGURE 11:**
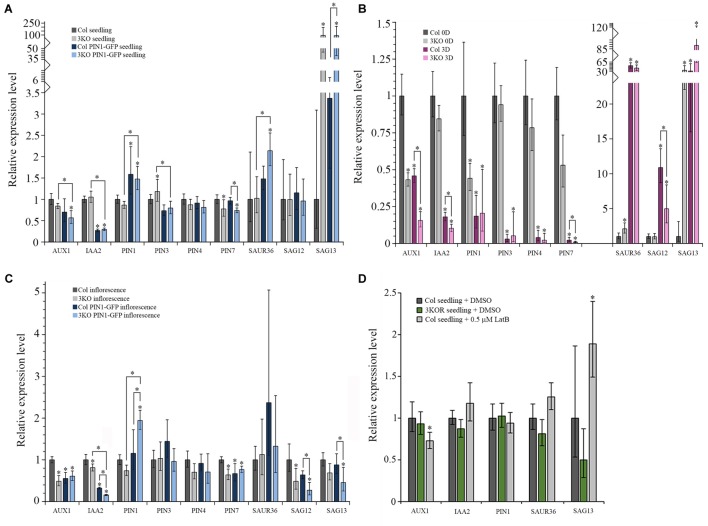
Relative mRNA expression levels of auxin-responsive genes and senescence-associated genes. **(A–D)** RT-qPCR analysis of *AUX1*, *IAA2*, *PIN1*, *PIN3*, *PIN4*, *PIN7*, *SAUR36*, *SAG12*, and *SAG13* mRNA levels in: **(A)** 7-day-old seedlings of Col (set to 1), 3KO, Col PIN1-GFP, and 3KO PIN1-GFP, *n* = 5–6; **(B)** leaves of 21-day-old rosettes of Col (0D set to 1) and 3KO before (0D) and after (3D) dark-induced senescence, *n* = 5–6; **(C)** inflorescences of mature stems of Col (set to 1), 3KO, Col PIN1-GFP, and 3KO PIN1-GFP, *n* = 5; **(D)** 7-day-old seedlings of untreated Col (set to 1), 3KOR, and 0.5 μM LatB-treated (6 h) Col, *n* = 6. Error bars represent 95% confidence intervals (CI); *^∗^p* < 0.05 (One-Way ANOVA).

First, the expression levels of selected auxin- and senescence-associated genes in the 3KO seedlings, leaves and inflorescences were compared with Columbia control. In the 7-day-old 3KO seedlings, early senescence-associated gene *SAG13* was 94-fold up-regulated when compared to the Columbia control (Figure 11A). The down-regulation of *PIN7* in mutant seedlings was moderate, although statistically significant (Figure 11A).

The relative levels of selected genes expressed in rosette leaves of 3KO and Columbia plants were compared before and after dark treatment. Before dark-treatment, at 21 days of growth, 3KO rosette leaves (3KO 0D) showed a 34-fold up-regulation of *SAG13* expression, when compared to untreated Columbia control (Col 0D) (Figure 11B). The expression of *SAUR36* in untreated 3KO (3KO 0D) leaves was twofold increased and those of *AUX1* and *PIN1* were twofold decreased, in comparison with untreated control leaves (Col 0D) (Figure 11B). The dark-treatment of detached leaves significantly affected the expression levels of all selected auxin-responsive and senescence-associated genes both in 3KO (3KO 3D) and Columbia (Col 3D) plants when compared to untreated control (Col 0D) (Figure 11B). However, the changes in 3KO background were more pronounced than those in Columbia. Down-regulation of *AUX1* (7-fold), *IAA2* (10-fold), and *PIN7* (110-fold) in dark-treated 3KO leaves was twice as high as in Columbia (Col 3D) (Figure 11B). It is noteworthy that the up-regulation of *SAG13* in dark-treated Columbia (Col 3D) was comparable with untreated 3KO leaves (3KO 0D), whereas in dark-treated mutant (3KO 3D), the *SAG13* was already 89-fold up-regulated (Figure 11B).

In 3KO inflorescences, levels of *AUX1* and *SAG12* were down-regulated twofold and those of *IAA2* and *PIN7* moderately, in comparison with Columbia control (Figure 11C). These results show that the onset of leaf senescence of 3KO plants is initiated at transcript level already in the 7-day-old seedlings, as demonstrated by the conspicuously high expression level of the *SAG13* gene. The unchanged levels of *SAG12* mRNA in seedlings and leaves indicates that the premature senescence of 3KO leaves most probably does not result from the activation of a developmental senescence program since the strong expression of *SAG13* suggests the stress-induced senescence and cell death. The relative expression levels of auxin-responsive *AUX1* and *PIN1* in 3KO were affected the most in leaves and inflorescences, implying that defective development of 3KO shoots could be influenced by reduced auxin responses.

The PIN1 is the principal member of the PIN family involved in development of aerial organs and the lead player in floral development (Gälweiler et al., 1998; Benková et al., 2003; Scarpella et al., 2006; Adamowski and Friml, 2015). Therefore, even the modest overexpression of PIN1 under native promoter is thought to affect plant phenotype. Therefore, the levels of auxin- and senescence-responsive genes in PIN1::PIN1-GFP expressing lines were compared with untransformed Columbia control. As we expected, both in 3KO PIN1-GFP, as well as in Col PIN1-GFP seedlings the expression of *PIN1* was about 1.5-fold up-regulated in comparison with Columbia control (Figure 11A). The level of *IAA2* was three–fourfold down-regulated in seedlings of PIN1::PIN1-GFP expressing Columbia and 3KO (Figure 11A). In 3KO PIN1-GFP seedlings, the level of *SAG13* was 92-fold up-regulated, similarly like in parental 3KO line (Figure 11A). Likewise, the level of *SAUR36* was also twofold up-regulated in 3KO PIN1-GFP seedlings compared to untransformed Columbia and 3KO (Figure 11A). A moderate down-regulation in levels of *AUX1*, *PIN3*, and *PIN7* in 3KO PIN1-GFP, and *PIN3* in Col PIN1-GFP seedlings was also detectable (Figure 11A).

In 3KO PIN1-GFP inflorescences, the expression of *PIN1* was up-regulated twofold, whereas in Col PIN1-GFP it was comparable with Columbia control (Figure 11C). The level of *IAA2* both in 3KO PIN1-GFP and Col PIN1-GFP inflorescences was down-regulated six and threefold, respectively (Figure 11C). Relative expression levels of *AUX1* and *PIN7* were down-regulated moderately in both PIN1-GFP expressing lines (Figure 11C). Senescence-associated genes, *SAG12* and *SAG13*, were four and twofold down-regulated in 3KO PIN1-GFP inflorescences, whereas down-regulation of *SAG12* in Col PIN1-GFP shoots was marginal (Figure 11C). These data show that even the mild overexpression of the major non-redundant member of the auxin efflux carrier family, *PIN1*, inevitably affects the expression of auxin-responsive and senescence-associated genes not only in 3KO, but in Columbia background too. These results indicate also that there is a mutual crosstalk between myosin-mediated transport, auxin-signaling and senescence-related processes.

Third, we investigated how the treatment with AF destabilizing drug latrunculin B (LatB) affects the expression levels of *AUX1*, *IAA2*, *PIN1*, *SAUR36*, and *SAG13* in 7-day-old Columbia seedlings in comparison with untreated Columbia and 3KOR seedlings (Figure 11D). Six hours after the application of 0.5 μM LatB the 1.9-fold up-regulation of *SAG13*, and moderate down-regulation of *AUX1* in Columbia seedlings was detectable (Figure 11D). In 3KOR seedlings, the levels of *AUX1*, *IAA2*, *PIN1, SAUR36*, and *SAG13* were comparable with untreated Columbia control (Figure 11D). These data show that the latrunculin B-mediated disruption of AFs activates the expression of stress-inducible *SAG13* gene, indicating that the actin cytoskeleton may be necessary for delivering stress-responses in plant cells.

## Discussion

Our results show that the three *Arabidopsis* class XI myosins, XI-1, XI-2, and XI-K, contribute to the auxin responses and cell death, and thereby affect developmental decisions during flower growth and leaf senescence. Using triple mutant line 3KO (Ojangu et al., 2012) and complemented line 3KOR (Peremyslov et al., 2012), we demonstrate that simultaneous depletion of these three myosins affects responsiveness of the auxin-dependent promoters and polarization of the PIN1 auxin efflux carrier, causes premature onset of senescence and cell death in leaves, elevates accumulation of anthocyanins, and changes the expression levels of genes related to these processes. We also show that the stable expression of myosin XI-K rescues the decreased fertility and prematurely senescent phenotype in 3KO background. This result implements that myosin XI-K plays important role not only in driving vegetative plant growth (Ojangu et al., 2007, 2012; Peremyslov et al., 2012) and gravitropic response (Talts et al., 2016), but contributes also to floral development and cell death.

Previous works on multiple gene knockout mutants have identified several myosin XI functions in plant development. In particular, the best studied phenotypes of 3KO plants included stunted growth, delayed bolting, incomplete development of stigmas and reduced fertility (Peremyslov et al., 2010; Ojangu et al., 2012). These phenotypic defects are explained with severe changes at the cellular level: disorganized and more static actin cytoskeleton, reduced membrane trafficking, nearly arrested cytoplasmic streaming (Peremyslov et al., 2010; Ueda et al., 2010; Cai et al., 2014), reduced sensitivity of vacuoles to exogenous auxin, and partial loss of PIN1 polarization in root cells (Scheuring et al., 2016; Abu-Abied et al., 2018).

First, we used flower development of 3KO plants as a model for examining myosin-dependent auxin-responsive processes in *Arabidopsis*. We show that the apical dominance and branching architecture of 3KO shoots is partially affected as the mutant plants produce more axillary branches on primary inflorescence stem. Investigating inflorescence development, four traits are usually evaluated: bolting time, length of the reproductive phase, number of rosette leaves at bolting, number of axillary branches and fruits (Ungerer et al., 2002; Pouteau and Albertini, 2009). In myosin 3KO mutant, all these developmental aspects are partly affected: bolting time delays, reproductive phase is expanded, more rosette leaves are formed, branching architecture is affected, and silique size is decreased. The branching architecture of inflorescence shoots is mainly regulated by auxin, cytokinin, and strigolactone, which control initiation and outgrowth of axillary meristem (Domagalska and Leyser, 2011). Basipetal auxin transport from shoot apex toward the base suppresses axillary meristem outgrowth, and leads to apical dominance (Davies et al., 1966). In *Arabidopsis* and tomato, the axillary meristem initiation is characterized by preparative auxin depletion and the subsequent meristem emergence by a local auxin accumulation (Wang et al., 2014). It has been showed that local auxin gradients necessary during phyllotactic patterning in leaf and inflorescence meristems are regulated by auxin importers together with PIN1 exporter (Bainbridge et al., 2008). Consistent with this, our results show that the auxin responsiveness is reduced throughout the development of the 3KO plants, with the most pronounced effects in seedlings and inflorescence stems. In addition, as the PIN1 polarization in epidermal cells of developing 3KO gynoecia is partially disturbed, and two major auxin transporter genes, *AUX1* and *PIN1*, are down-regulated in 3KO leaves and inflorescences, a correlation between these processes can be found, indicating that PAT-related processes are disturbed in 3KO background. Interestingly, our results complement recently published data which demonstrated that the production of lateral and adventitious roots in 3KO plants was increased (Abu-Abied et al., 2018). The abnormal formation of the lateral roots was related to changed auxin gradient, which was in correlation with partial loss of PIN1 polarization in stele cells of 3KO plants. Moreover, authors revealed the myosin XI-K role in cell division in both the root and shoot meristem (Abu-Abied et al., 2018). The reduced auxin responsiveness and loss of myosin XI-K function in 3KO meristem was used to explain the altered branching architecture of mutant roots (Abu-Abied et al., 2018). Although these two studies (current work; Abu-Abied et al., 2018) used different models (flowers and leaves versus roots) and different experimental approaches, both highlight myosin-auxin connections.

In this work, we demonstrate for the first time that the myosin XI-K:YFP is expressed in floral primordia and in developing flowers, indicating its role in floral development. Moreover, correlation between the expression pattern of XI-K:YFP and the delayed elongation of stigmas and anther filaments in 3KO can be found, though we do not exclude the roles of myosin XI-1 and XI-2 in these processes.

It is broadly assumed that development of the stamens and gynoecia, the most modified floral organs, require enhanced production of hormones, including auxin (Okada et al., 1991; Sessions et al., 1997; Nemhauser et al., 2000; Aloni et al., 2006; Cheng et al., 2006; Cecchetti et al., 2008; Sundberg and Østergaard, 2009; Hawkins and Liu, 2014). It is proposed that the finely tuned auxin gradient is necessary for gynoecium patterning in general and style and stigma development in particular (Nemhauser et al., 2000). To complete the development of valves, style and stigma, the medial, lateral and apical domains of the growing gynoecium are provided with auxin from the base. This has been demonstrated by Larsson et al. (2014), who showed that the PIN1-GFP localization in outer epidermal cells of stage 7 gynoecia was prominently apical. Interestingly, we found that stable expression of the PIN1::PIN1-GFP augmented a semi-sterile phenotype of the 3KO plants (Peremyslov et al., 2010; Ojangu et al., 2012). Our results indicate that the ability of 3KO PIN1-GFP inflorescence meristem to produce flowers with determined number and shape of inner whorl organs is affected to a variable degree, whereas the gynoecium development is affected the most. Similar gynoecium phenotypes have been described upon direct genetic or pharmaceutical disruption of auxin signaling (Sessions et al., 1997; Nemhauser et al., 2000; Cheng et al., 2006; Zúñiga-Mayo et al., 2014). The gynoecium is the last organ which emerges from the floral meristem (Larsson et al., 2014), and the style and stigma are last structures which emerge from the growing gynoecium. The partial loss of PIN1 polarization in developing 3KO PIN1-GFP gynoecia may indicate that the auxin transport from the base may not be sufficient to complete the growth of style, stigma or valves of 3KO PIN1-GFP pistils correctly. Here we demonstrate also that the late phase of stamen development, pre-anthesis filament elongation, often delays both in the parental line as well as in 3KO PIN1-GFP line. Our results indicate that the defective floral development of the 3KO PIN1-GFP line could be due to inability of the disrupted actomyosin cytoskeleton to properly allocate an excess of PIN1 which in turn, via feedback signaling pathway, may affect the expression of auxin-responsive genes. All these results together indicate the possible cooperation between myosin-mediated trafficking and auxin responses in floral development.

Second, we used leaf development of 3KO plants as a model for examining myosin-dependent senescence-associated processes in *Arabidopsis*. It is well documented that late flowering plants have more rosette leaves, and that there is a strong correlation between leaf number and bolting time (Pouteau and Albertini, 2009; Schmalenbach et al., 2014). In accordance with this, rosettes of 3KO plants create more leaves, but at the same time show premature signs of leaf senescence. Premature senescence in 3KO leaves is confirmed by massive loss of chlorophyll, enhanced cell death, premature rupture of petiole epidermal cells, and abnormal accumulation of anthocyanins. Our finding that the *SAG13* is significantly up-regulated prior to observable signs of leaf yellowing both in young 3KO seedlings as well as in rosette leaves, indicates that the premature senescence in this mutant line is highly accelerated at transcript level. In addition, the expression of late senescence-associated gene *SAG12* is not different in the 3KO seedlings and leaves relative to the Columbia. Moreover, the accumulation of anthocyanins in 3KO plants is in accordance with the fact that increased flavonoid production (e.g., anthocyanins) has been also associated with stress responses (Solfanelli et al., 2006). As we detect the anthocyanin accumulation, and *SAG13* up-regulation already in young 3KO seedlings, this supports our assumption that the early leaf senescence of 3KO mutants is not typical developmental senescence but could be caused by cellular stresses such as changes in membrane trafficking, AF rearrangement, and auxin responses. For example, when wild type *Arabidopsis* seedlings are suffering from salt and methyl jasmonate co-stress, the leaf senescence is prematurely initiated as the *SAG13* of transcript level is significantly up-regulated (Chen et al., 2017). In *Arabidopsis accelerated-cell-death11* (*acd11*) mutant, the *SAG13* mRNA is strongly expressed, whereas *SAG12* mRNA does not accumulate. The authors concluded that the cell death in *acd11* does not result from activation of a senescence program, but from PCD as suggested by conspicuously strong *SAG13* expression (Brodersen et al., 2002). It is tempting to speculate that in 3KO cells, the loss of integrity of actomyosin cytoskeleton activates the stress-signaling pathway, and as a consequence of this the senescence and cell death are prematurely initiated.

It has been shown that the integrity of actin cytoskeleton contributes to senescence and cell death through reorganization of the endoplasmic reticulum (ER) network (Boevink et al., 1998; Kasaras et al., 2012; Xu et al., 2012; Carvalho et al., 2014; Chang et al., 2015) and transcription factor activation (Hao and August, 2005; Breeze et al., 2011; Smertenko and Franklin-Tong, 2011). Smertenko et al. (2003) suggested that the filamentous actin is crucial for the developmentally programmed cell death in the Norway spruce embryos as AFs disappeared significantly later than the microtubules. It has been proposed also that during leaf senescence, a functional actin cytoskeleton could be essential for maintaining the primary metabolism until the cell death (Keech, 2011). Because the integrity of actin arrays, and thus membrane trafficking is affected in 3KO epidermal cells (Peremyslov et al., 2010; Cai et al., 2014), a causal connection between these processes can be suggested. Myosin’s potential role in senescence is supported by the facts that ER-streaming is dramatically suppressed in 3KO plants (Ueda et al., 2010), and a portion of the XI-K:YFP is aligned and co-fractionated with a motile ER subdomain (Peremyslov et al., 2012). Intriguingly, myosins XI-K and XI-2 that are inactivated in 3KO are not only broadly expressed throughout plant tissues, but show particularly high expression level in the senescent leaves too (Peremyslov et al., 2011).

In general, the role of auxin in regulating leaf senescence still remains elusive. It has been implied that auxin may promote leaf senescence via *SAUR36* gene since the auxin-inducible transcript accumulation promotes premature senescence of young leaves, whereas in *saur36* null mutant plants senescence delays substantially (Hou et al., 2013). Our findings show that the premature leaf senescence of 3KO leaves is indeed accompanied by up-regulation of *SAUR36*, although it is not clear how the early aging is influenced by reduced auxin responses in this mutant. Up-regulation of *SAG13* and *SAUR36* during leaf senescence, regardless of whether it is induced naturally or by darkness suggests that the senescence mechanism under these two different conditions share common features. It is well known that the dark-induced senescence of detached leaves is radical intervention as major changes in leaf physiology inevitably affect the gene expression. Although, the dark-treatment of detached 3KO leaves does not reflect the situation of natural senescence, the results obtained from these experiments, provide additional clues that myosins contribute to the auxin- and senescence-responsive processes, and indicate that these processes are mutually interconnected.

Accumulation of anthocyanins in plant tissues is related both with auxin signaling and senescence (Brown et al., 2001; Feild et al., 2001; Buer and Muday, 2004; Besseau et al., 2007; Schippers et al., 2007; Falcone Ferreyra et al., 2012). In particular, auxin transport is elevated in inflorescences, hypocotyls, and roots of plants of the flavonoids-deficient mutants (Murphy et al., 2000; Brown et al., 2001; Lewis et al., 2011). During senescence, anthocyanins reduce the risk of photo-oxidative damage in leaf cells and thereby help retrieve nutrients from senescing tissues (Feild et al., 2001; Falcone Ferreyra et al., 2012). We propose that the accumulation of anthocyanins in 3KO tissues is an accompanying effect influenced both by reduced auxin responses as well as by premature onset of senescence. Collectively, this work highlights the importance of actomyosin cytoskeleton in auxin responsiveness and senescence, and leads to further questions as to how reorganization of metabolism is achieved in cells having a drastically affected actomyosin cytoskeleton. The next critical step in understanding these networks is investigation of the mechanistic contributions of myosins to the function of these networks. It is anticipated that such contributions could involve specific role of myosin-dependent transport in PIN and AUX targeting, overall reduction in cytoplasmic streaming that could affect auxin transport or auxin diffusion, as well as myosin-dependent changes in AF organization. Whether these myosins contribute to auxin responses through shaping actin cytoskeleton or via reorganization of the ER remains to be elucidated. Important aspect of the future studies will be identifying the myosin cargoes that affect auxin signaling and senescence. These cargoes could include MyoB myosin receptors that appear to drive cytoplasmic streaming (Peremyslov et al., 2013, 2015) or newly identified myosin adaptors of MadA and MadB families that presumably mediate more specialized myosin-dependent processes (Kurth et al., 2017).

**FIGURE 12:**
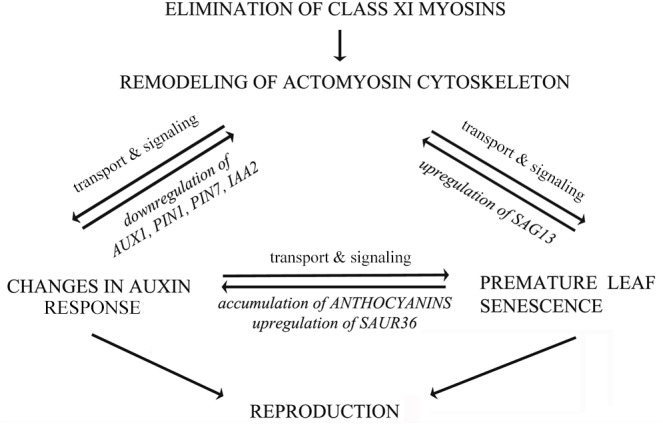
Hypothetical model illustrating mutual influences between actomyosin cytoskeleton, auxin transport and leaf senescence. The actomyosin cytoskeleton mediated signaling of auxin responses involves both the distribution of auxin efflux carrier PIN1, as well as the regulation of the expression of auxin-responsive genes. The actomyosin cytoskeleton mediated signaling of senescence and cell death involves the control of expression of stress-inducible senescence-associated gene *SAG13*. Senescence, cell death and auxin signaling could be mutually related via secondary metabolism and expression of auxin-responsive *SAUR36* gene. The auxin responses and leaf senescence could affect inflorescence biomass production in opposite directions. The bidirectional arrows imply mutual influences between these processes, indicating that if one is unbalanced the others will be affected to various degrees.

Taken together, our results provide the genetic evidence that the integrity of actomyosin cytoskeleton, signaling of auxin and senescence, as well as secondary metabolism are functionally intertwined in a finely tuned network as illustrated in a tentative model (Figure 12). According to this, remodeling of actomyosin cytoskeleton affects not only distribution of auxin exporter PIN1, but also the expression of auxin-responsive and senescence-associated genes. Most likely, the levels of auxin-responsive genes are influenced indirectly, through feedback signaling mechanism, mediated by changes in auxin responses. The actomyosin cytoskeleton-mediated signaling of premature leaf senescence involves the control of expression of stress-inducible gene *SAG13*. Senescence and auxin signaling could be mutually regulated by secondary metabolism (e.g., anthocyanin accumulation) and by expression of auxin-responsive *SAUR* genes (e.g., *SAUR36*). All these processes are mutually interconnected, and if one process is unbalanced the others are affected too. It is tempting to speculate that changes in auxin and senescence responses influence reproduction of 3KO plants in opposite directions (Figure 12). From the one hand, the reduced auxin response could disturb flower development; on the other hand, it seems that the premature leaf senescence could constitute a rescue mechanism for supporting the production of inflorescence biomass in conditions where flower development has been compromised.

## Author Contributions

E-LO, BI, KTal, HP, ET, and VD contributed to design of the study and interpretation of data. E-LO, BI, KTal, KTan, and EI conducted experiments and analyzed data. KTan performed anthocyanin and chlorophyll measurements, and trypan blue stainings. BI performed RT-qPCR. EI performed histochemical GUS stainings. KTal helped with protocol optimization for quantitative MUG assay. HP helped with SEM and confocal imaging, image processing, and data analysis. VD generated 3KOR line. E-LO, BI, KTal, HP, VD, and ET drafted the manuscript and revised it critically. All authors read and approved the final manuscript, and they agreed to be accountable for all aspects of the work.

## Conflict of Interest Statement

The authors declare that the research was conducted in the absence of any commercial or financial relationships that could be construed as a potential conflict of interest.
