# Supplementary material for: Class XI Myosins Contribute to Auxin Response and Senescence-Induced Cell Death in Arabidopsis
Source: Front Plant Sci. 2018 Nov 27;9:1570. doi: 10.3389/fpls.2018.01570 (PMC6277483; doi:10.3389/fpls.2018.01570)
Supplement: Supplementary file 1 [file Table_1.pdf]

Supplementary Material

Class XI myosins contribute to auxin response and senescence-induced cell death in Arabidopsis

Eve-Ly Ojangu*, Birger Ilau, Krista Tanner, Kristiina Talts, Eliis Ihoma, Valerian V. Dolja, Heiti Paves, Erkki Truve

*** Correspondence:** Eve-Ly Ojangu: eve-ly.ojangu@ttu.ee

1 Supplementary Table

Supplementary Table S1. RT-qPCR primers.

Gene name	AGI code	Primer sequence
SAND	AT2G28390	5’ - AACTCTATGCAGCATTTGATCCACT - 3’
		5’ - TGATTGCATATCTTTATCGCCATC - 3’
UBC	AT5G25760	5’ - CTGCGACTCAGGGAATCTTCTAA - 3’
		5’ - TTGTGCCATTGAATTGAACCC - 3’
expressed sequence (*EX70*)	AT2G32170	5’ - ATCGAGCTAAGTTTGGAGGATGTAA - 3’
		5’ - TCTCGATCACAAACCCAAAATG - 3’
AUX1	AT2G38120	5’- TGTTATCAGGAATAGTACTTCAGATC - 3’
		5’- AGTATGAACCAAGTAATCCATCAAG - 3’
IAA2	AT3G23030	5’- AGAACAACAACAGTGTGAGCTAC - 3’
		5’- CTCTCACAATATTCACCAATCATGA - 3’
PIN1	AT1G73590	5’- CGACACTCCCCAACACTCTAG - 3’
		5’- AGCTTAGCTCCACGGTACTC - 3’
PIN3	AT1G70940	5’- AAAGATTGGAAGATGAAGACAACTTA - 3’
		5’- CTGGAACAAGGGAATATTCAAAATC - 3’

PIN4	AT2G01420	5'- GGATCTTGTCTTCATTAGACGC - 3'
		5'- GTATAAACCACTTAACTAGAAACTTCA - 3'
PIN7	AT1G23080	5'- TCGCACTACCGATTACACTTGTT - 3'
		5'- CATTTCGTGTTACATTACATTTTCTAG - 3'
SAUR36	AT2G45210	5'- GTGATTAGCATGGGGACACTTA - 3'
		5'- ATCTTGAGTAGACCCATATAGAGAA - 3'
SAG12	AT5G45890	5'- TGATGAGCAAGCACTGATGAAG - 3'
		5'- TTCGCCGTATCCAATCGCAG - 3'
SAG13	AT2G29350	5'- AGCTTGCCCACCCATTGTTAAA - 3'
		5'- CCAGCTGATTCATGGCTCCT - 3'
