# Supplementary material for: Class XI Myosins Contribute to Auxin Response and Senescence-Induced Cell Death in Arabidopsis
Source: Front Plant Sci. 2018 Nov 27;9:1570. doi: 10.3389/fpls.2018.01570 (PMC6277483; doi:10.3389/fpls.2018.01570)
Supplement: Supplementary file 2 [file Image_1.pdf]

Supplementary Material

Class XI myosins contribute to auxin response and senescence-induced cell death in Arabidopsis

Eve-Ly Ojangu*, Birger Ilau, Krista Tanner, Kristiina Talts, Eliis Ihoma, Valerian V. Dolja, Heiti Paves, Erkki Truve

*** Correspondence:** Eve-Ly Ojangu: eve-ly.ojangu@ttu.ee

1 Supplementary figure 1

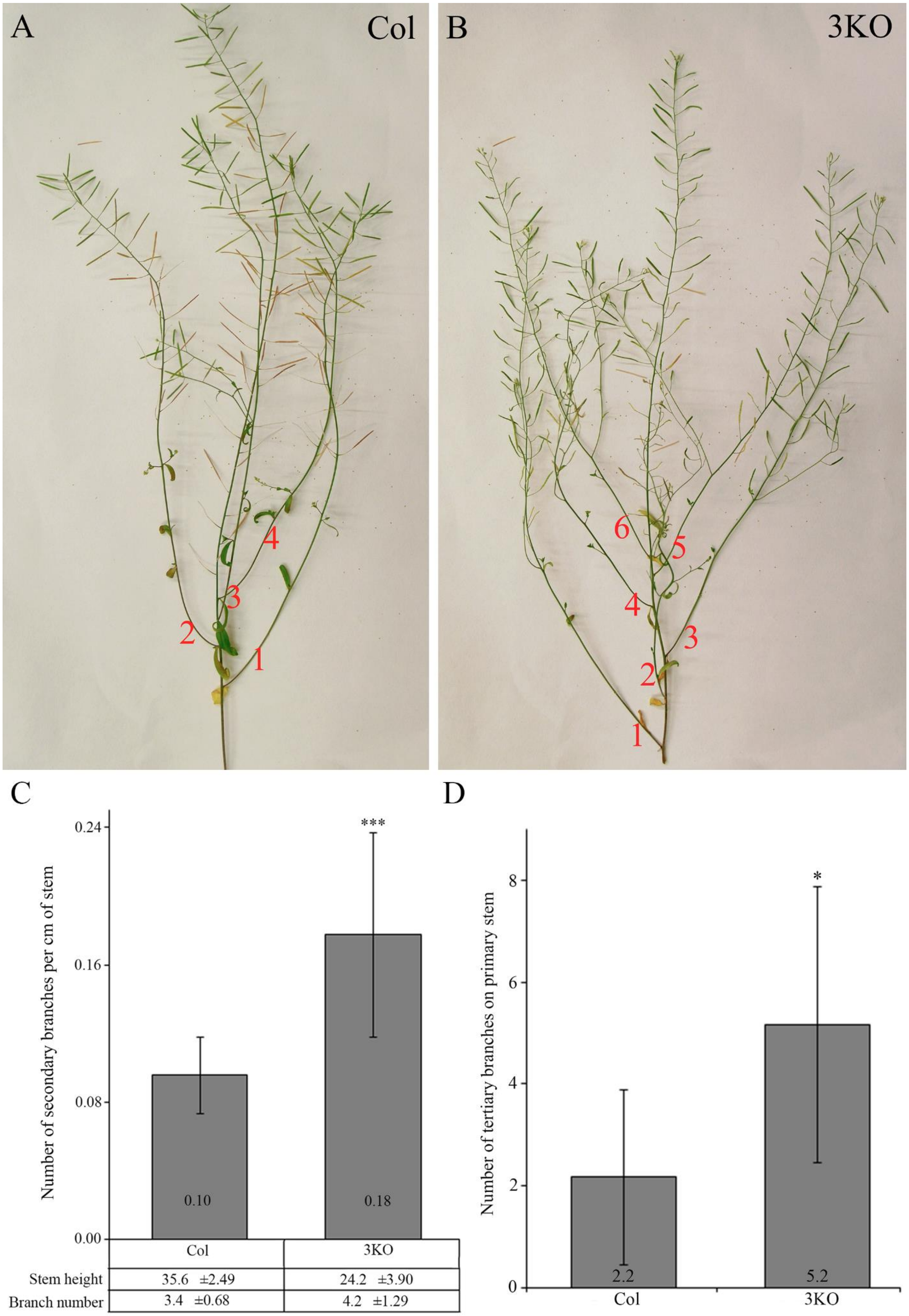
A
Col
4
3
2
1
B
3KO
6
5
4
3
2
1
C
Number of secondary branches per cm of stem
0.24
0.16
0.08
0.00
***
0.10
0.18
Col
3KO
Stem height
35.6 ±2.49
24.2 ±3.90
Branch number
3.4 ±0.68
4.2 ±1.29
D
Number of tertiary branches on primary stem
8
6
4
2
0
*
2.2
5.2
Col
3KO

Supplementary Figure 1. Branching architecture of primary inflorescence shoots. (A) Primary inflorescence shoot of Col plant formed four axillary branches, and 3KO plant **(B)** six axillary branches. Red numbers illustrate the number of axillary branches. **(C)** Number of secondary branches per cm of primary inflorescence stem. Stem height and branch number was measured after flower formation was ended. Data represent average values; error bars represent SD; n = 17-19; *** $p < 0.001$ (Student's *t*-test). **(D)** Number of tertiary branches on primary inflorescence stem. Data represent average values; error bars represent SD; n = 6; * $p < 0.05$ (Student's *t*-test).
