# Supplementary material for: Class XI Myosins Contribute to Auxin Response and Senescence-Induced Cell Death in Arabidopsis
Source: Front Plant Sci. 2018 Nov 27;9:1570. doi: 10.3389/fpls.2018.01570 (PMC6277483; doi:10.3389/fpls.2018.01570)
Supplement: Supplementary file 3 [file Image_2.pdf]

Supplementary Material

Class XI myosins contribute to auxin response and senescence-induced cell death in Arabidopsis

Eve-Ly Ojangu*, Birger Ilau, Krista Tanner, Kristiina Talts, Eliis Ihoma, Valerian V. Dolja, Heiti Paves, Erkki Truve

*** Correspondence:** Eve-Ly Ojangu: eve-ly.ojangu@ttu.ee

1 Supplementary figure 2

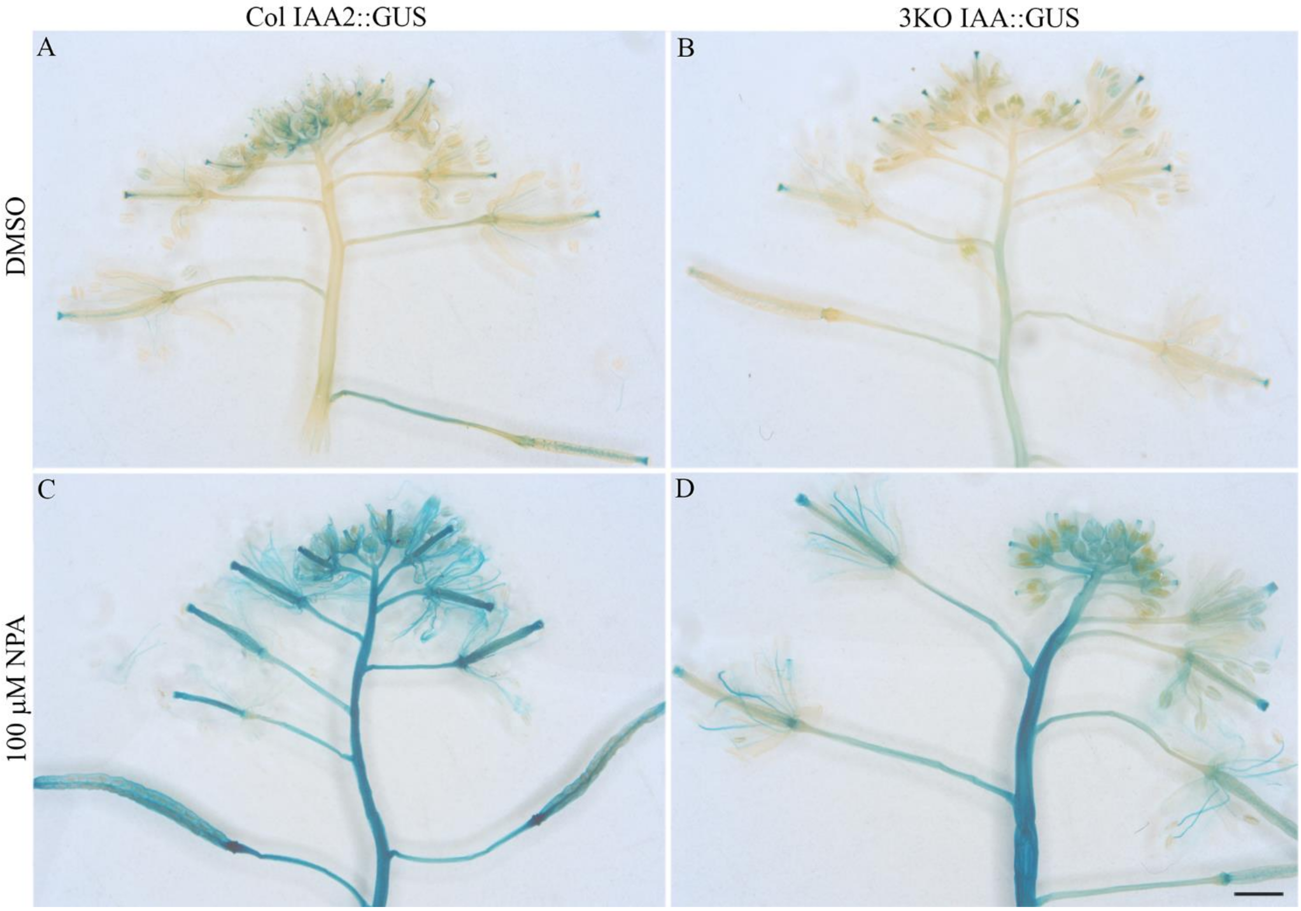

Supplementary Figure 2. Effect of NPA-treatment on IAA2::GUS expression. Primary inflorescences of Col IAA2::GUS **(A)** and 3KO IAA2::GUS **(B)** were dipped twice with 100 µM NPA **(C-D)**. Histochemical staining was performed one week after NPA-treatment. NPA-treatment led to

increased IAA2::GUS activity both in Col **(C)** and 3KO **(D)** inflorescences. In NPA-treated 3KO IAA2::GUS flowers, the GUS staining does not accumulate in valves of gynoecia. Somewhat weaker staining of pedicles, petals and sepals is noticeable in 3KO IAA2::GUS also. Scale bar is 1 mm.
