# Supplementary material for: Class XI Myosins Contribute to Auxin Response and Senescence-Induced Cell Death in Arabidopsis
Source: Front Plant Sci. 2018 Nov 27;9:1570. doi: 10.3389/fpls.2018.01570 (PMC6277483; doi:10.3389/fpls.2018.01570)
Supplement: Supplementary file 4 [file Image_3.pdf]

Supplementary Material

Class XI myosins contribute to auxin response and senescence-induced cell death in Arabidopsis

Eve-Ly Ojangu*, Birger Ilau, Krista Tanner, Kristiina Talts, Eliis Ihoma, Valerian V. Dolja, Heiti Paves, Erkki Truve

*** Correspondence:** Eve-Ly Ojangu: eve-ly.ojangu@ttu.ee

1 Supplementary figure 3

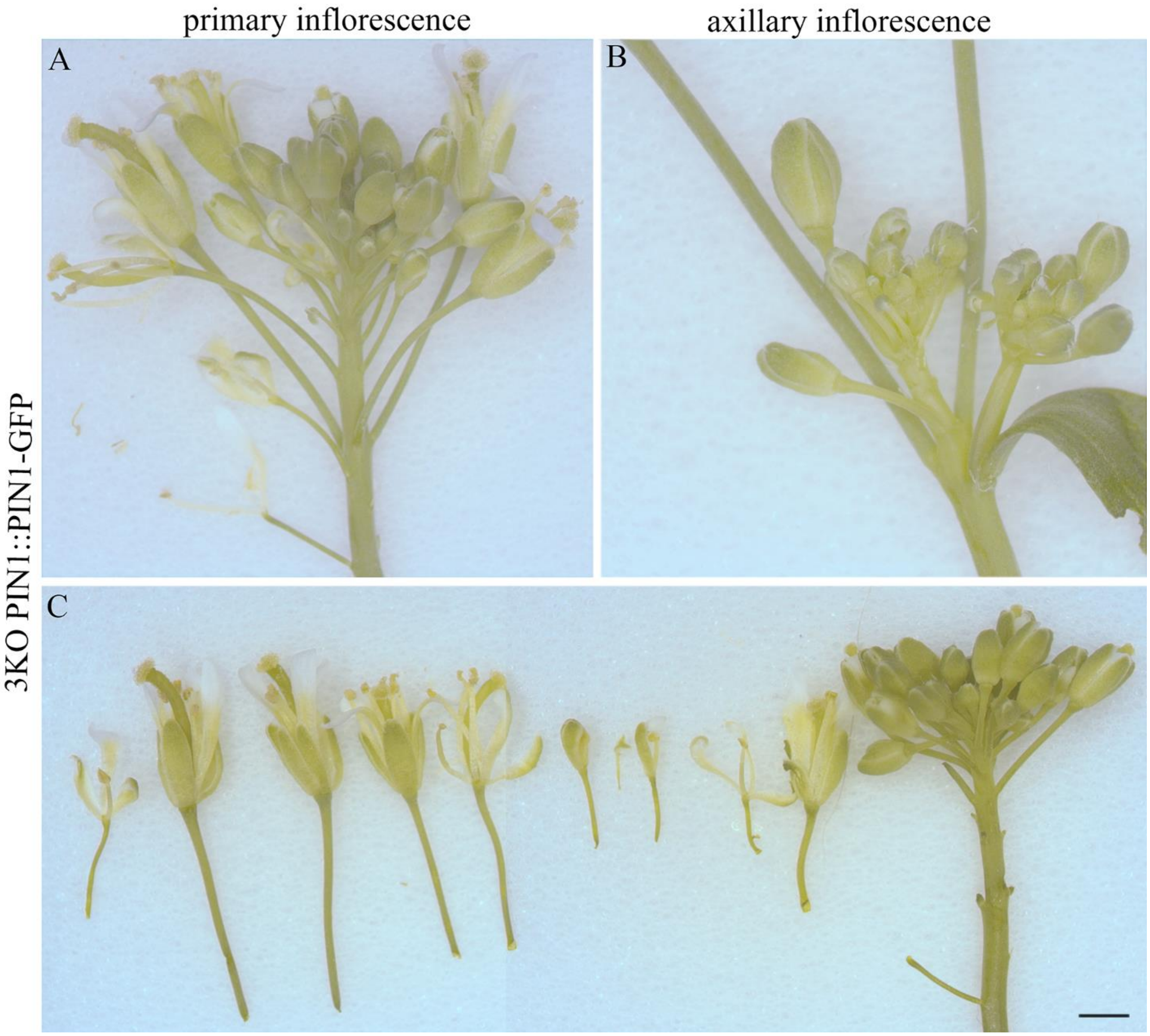

Supplementary Figure 3. Variety of inflorescence and flower development in 3KO PIN1::PIN1-GFP plants. The irregular architecture of **(A)** primary inflorescence and **(B)** axillary (secondary) inflorescence of 3KO PIN1::PIN1-GFP plants. **(C)** Post-anthesis flowers were dissected from primary inflorescence **(A)** according to developmental sequence. Notice the variability of under- and undeveloped flowers. Scale bar is 1mm.
