# Supplementary material for: Class XI Myosins Contribute to Auxin Response and Senescence-Induced Cell Death in Arabidopsis
Source: Front Plant Sci. 2018 Nov 27;9:1570. doi: 10.3389/fpls.2018.01570 (PMC6277483; doi:10.3389/fpls.2018.01570)
Supplement: Supplementary file 5 [file Image_4.pdf]

Supplementary Material

Class XI myosins contribute to auxin response and senescence-induced cell death in Arabidopsis

Eve-Ly Ojangu*, Birger Ilau, Krista Tanner, Kristiina Talts, Eliis Ihoma, Valerian V. Dolja, Heiti Paves, Erkki Truve

*** Correspondence:** Eve-Ly Ojangu: eve-ly.ojangu@ttu.ee

1 Supplementary figure 4

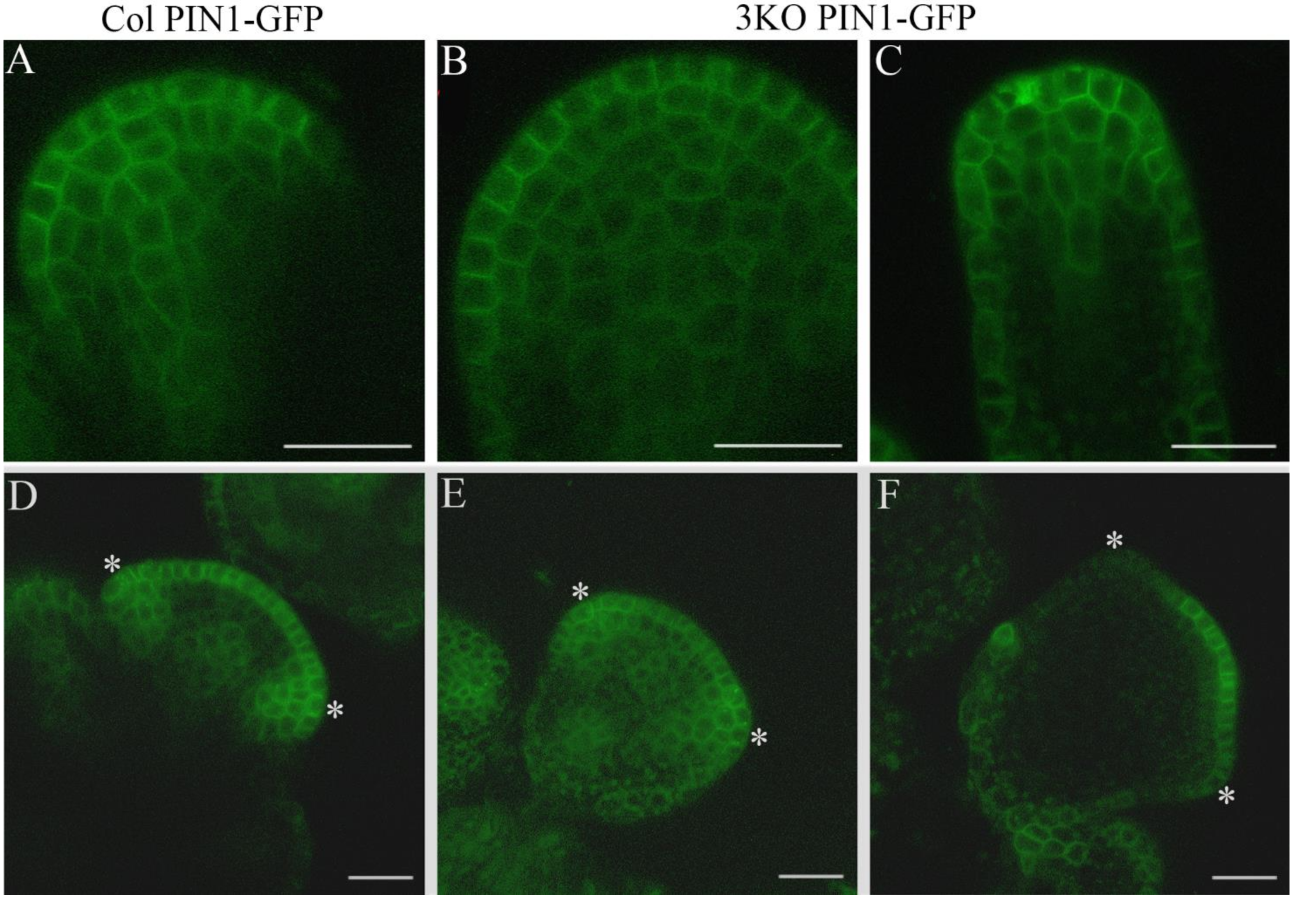

Supplementary Figure 4. The PIN1-GFP expression in early stages of floral primordia development. **(A-C)** Upper panel represents very early stage of primordium development. In 3KO PIN1-GFP plants, the shapes of primordia vary occasionally, notice the pin-like shape of 3KO PIN1-GFP primordium **(C)**. **(D-F)** Lower panel represents later stage of primordium development where PIN1-GFP accumulation defines the sites of petal initiation (white asterisks). The PIN1-GFP patterning in developing 3KO floral primordia is partially aberrant since the PIN1 accumulation is occasionally absent at the sites of petal emergence (**F**). Images represent single optical slices. Scale bars are 20 μm.
