# Supplementary material for: Class XI Myosins Contribute to Auxin Response and Senescence-Induced Cell Death in Arabidopsis
Source: Front Plant Sci. 2018 Nov 27;9:1570. doi: 10.3389/fpls.2018.01570 (PMC6277483; doi:10.3389/fpls.2018.01570)
Supplement: Supplementary file 6 [file Image_5.pdf]

Supplementary Material

Class XI myosins contribute to auxin response and senescence-induced cell death in Arabidopsis

Eve-Ly Ojangu*, Birger Ilau, Krista Tanner, Kristiina Talts, Eliis Ihoma, Valerian V. Dolja, Heiti Paves, Erkki Truve

*** Correspondence:** Eve-Ly Ojangu: eve-ly.ojangu@ttu.ee

1 Supplementary figure 5

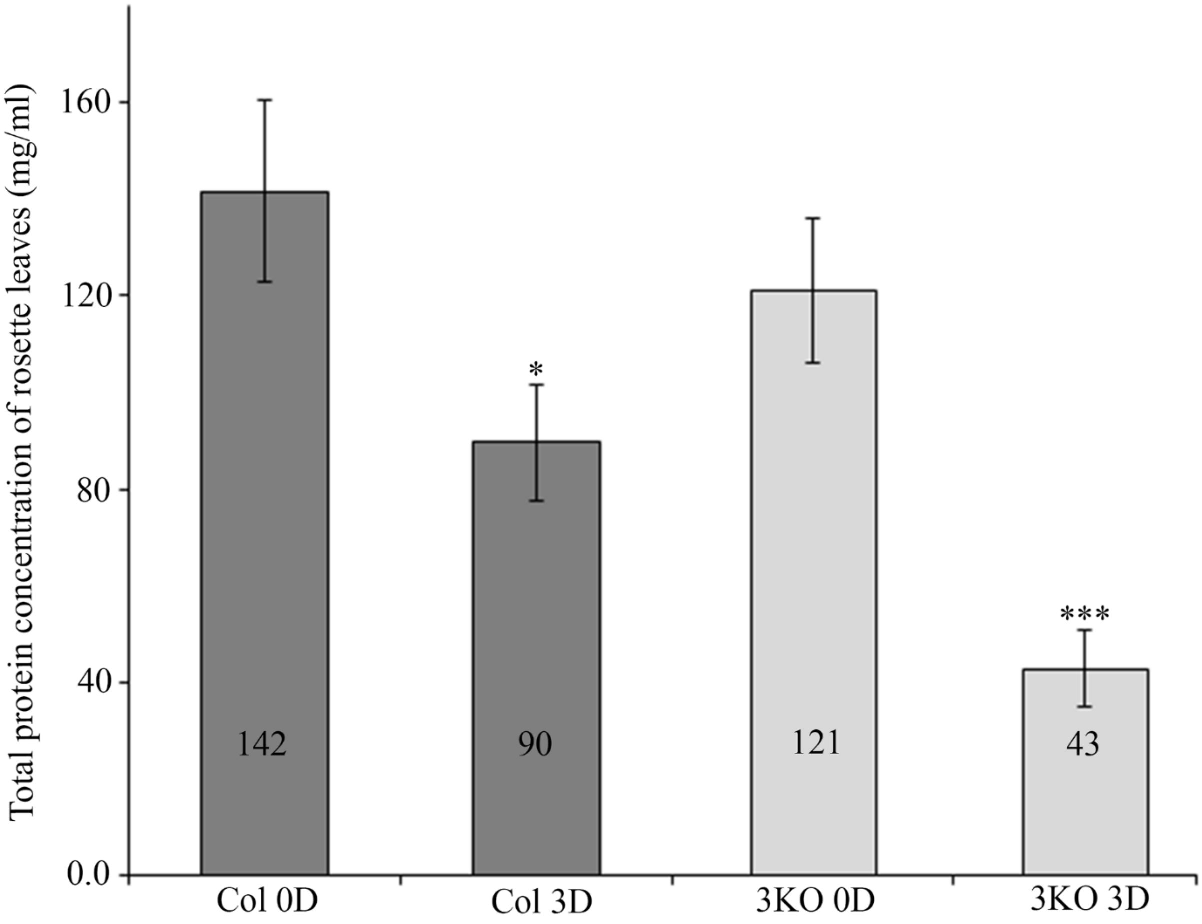

Supplementary Figure 5. The total protein content of leaf extracts before and after dark treatment. After dark-treatment, the total protein concentration of Col leaves was 1.6-fold lower (Col 3D) and the one of 3KO leaves was 2.8-fold lower (3KO 3D), in comparison with untreated controls

(Col 0D, 3KO 0D), respectively. Data represent average values; error bars represent SD; n = 4; * $p < 0.05$, *** $p < 0.001$ (Student's *t*-test).
